# RNA methylation in nuclear pre‐mRNA processing

**DOI:** 10.1002/wrna.1489

**Published:** 2018-06-19

**Authors:** Helena Covelo‐Molares, Marek Bartosovic, Stepanka Vanacova

**Affiliations:** ^1^ CEITEC, Masaryk University Brno Czech Republic

**Keywords:** RNA demethylase, RNA methylase, RNA processing

## Abstract

Eukaryotic RNA can carry more than 100 different types of chemical modifications. Early studies have been focused on modifications of highly abundant RNA, such as ribosomal RNA and transfer RNA, but recent technical advances have made it possible to also study messenger RNA (mRNA). Subsequently, mRNA modifications, namely methylation, have emerged as key players in eukaryotic gene expression regulation. The most abundant and widely studied internal mRNA modification is N^6^‐methyladenosine (m^6^A), but the list of mRNA chemical modifications continues to grow as fast as interest in this field. Over the past decade, transcriptome‐wide studies combined with advanced biochemistry and the discovery of methylation writers, readers, and erasers revealed roles for mRNA methylation in the regulation of nearly every aspect of the mRNA life cycle and in diverse cellular, developmental, and disease processes. Although large parts of mRNA function are linked to its cytoplasmic stability and regulation of its translation, a number of studies have begun to provide evidence for methylation‐regulated nuclear processes. In this review, we summarize the recent advances in RNA methylation research and highlight how these new findings have contributed to our understanding of methylation‐dependent RNA processing in the nucleus.

This article is categorized under:

RNA Processing > RNA Editing and Modification
RNA Processing > Splicing Regulation/Alternative Splicing
RNA Interactions with Proteins and Other Molecules > Protein–RNA Interactions: Functional Implications

## 1 INTRODUCTION

Posttranscriptional processing of messenger RNA (mRNA) is a common feature of eukaryotes. The 5′ capping and 3′ polyadenylation modifications were discovered almost 50 years ago and have remained as one of the hallmarks of eukaryotic mRNA processing (Edmonds, Vaughan, & Nakazato, 1971; Muthukrishnan, Both, Furuichi, & Shatkin, 1975; C. M. Wei, Gershowitz, & Moss, 1975b). However, the recent discovery of additional internal, functional mRNA modifications has introduced a previously unappreciated level of mRNA metabolism regulation. This review will discuss the latest development in the field of mRNA modifications from the perspective of nuclear mRNA processing.

The pioneering analysis of methylated nucleotides in mammalian mRNA in the 1970s revealed the presence of a 7‐methylguanosine (m^7^G) cap at the 5′ end. It also unexpectedly detected internal methylated nucleotides (Desrosiers, Friderici, & Rottman, 1974; Perry & Kelley, 1974); N^6^‐methyladenosine (m^6^A) was the most abundant modification (Dubin & Taylor, 1975; Salditt‐Georgieff et al., 1976). These studies provided valuable insights into the composition and proportions of various internal mRNA modifications. However, they lacked thorough biochemical and functional analyses due to technical limitations. The big boom in the field came with the development of high‐throughput sequencing methods combined with biochemistry and/or specific antibodies, which provided tools to obtain a whole‐transcriptome view of RNA modifications. Individual RNA modifications can be detected by specific approaches. Some may be directly detected as mismatch mutations (e.g., A to I or C to U editing) or they must first be chemically modified to induce a mutation (e.g., bisulfite‐based sequencing of m^5^C). In other cases, such as N^1^‐methyladenosine (m^1^A) or m^6^A, the detection methods either rely on interference with reverse transcriptase or utilization of specific antibodies (Dominissini et al., 2012; Meyer et al., 2012; Tserovski et al., 2016). The recognition of the biological importance of RNA modifications, along with methodical developments, has led to “epitranscriptomics.” This field, analogous to epigenetics, studies functionally relevant chemical RNA modifications that do not alter the genomic sequence.

Thanks to methodological and technical developments, several major discoveries have changed our view on the importance of RNA modifications. In addition to reversible chemical modifications of DNA and proteins, RNA marks (mainly adenosine methylations) constitute yet another layer of gene expression regulation. In diverse eukaryotic lineages, dynamic RNA methylation plays a key role in such elementary processes as germline maturation (Batista et al., 2014; Geula et al., 2015; Y. Wang et al., 2014), early embryogenesis (Ivanova et al., 2017; Zhao et al., 2017), host defense against pathogens (Martinez‐Perez et al., 2017; Tirumuru et al., 2016), and cancer self‐renewal and tumorigenesis (Cui et al., 2017; Z. Li et al., 2017; Zhang et al., 2017) (Box 1). On the cellular level, methylation regulates a wide range of RNA‐related processes: RNA processing, stability, and translation. The aim of this review is to discuss the function of RNA methylation in nuclear pre‐mRNA processing. Most of the recent discoveries have been made in mammalian systems, but there is also new data from other eukaryotes, such as yeast, flies, and plants (Schwartz et al., 2013; Zhang et al., 2015). The spectrum of newly identified mRNA modifications grows every year; however, only a few have been studied in such detail as to be able to draw conclusions about their function. We will first discuss the growing spectrum of mRNA methylation marks, describe the known machinery involved in writing, erasing, and reading methylated mRNA, and finally we will discuss how these marks are involved in the early steps of mRNA processing in the nucleus.

BOX 1 **m^6^A IN DEVELOPMENT AND CANCER**

Several studies have indicated that m^6^A is a key regulator during early development and gametogenesis in different species. In mammals, m^6^A controls the stability of pluripotency regulators and thus allows embryonic stem cell (ESC) differentiation into specific cell types (Batista et al., 2014; Geula et al., 2015; Y. Wang, Li, et al., 2014). Deletion of the m^6^A writer, the Methyltransferase‐Like 3 (METTL3) in (KO) mouse ESCs is viable, but they failed to differentiate into specific lineages. Mettl3 KO in mouse is embryonically lethal (Geula et al., 2015). Failure of embryonic development was also observed in *Arabidopsis thaliana* upon depletion of the METTL3 ortholog (Zhong et al., 2008). *Drosophila* embryos were viable, but they suffered from severe neurological defects and reduced fertility (Haussmann et al., 2016; Lence et al., 2016). Moreover, the m^6^A eraser AlkB Homolog 5 (ALKBH5) was required for mouse spermatogenesis (Zheng et al., 2013), and the m^6^A readers YTHD Domain Family 2 (YTHDF2) and YTH Domain Containing 2 (YTHDC2) were needed for early zygote development in mammals and zebrafish and successful meiotic program in the mammalian germline, respectively (Hsu et al., 2017; Ivanova et al., 2017; Wojtas et al., 2017; Zhao et al., 2017; Box 2). Emerging evidence also suggests that m^6^A could play a role in cancer. For instance, the m^6^A methylation/demethylation pathway regulated glioblastoma stem cell self‐renewal and tumorigenesis (Cui et al., 2017; Zhang et al., 2017), and METTL3, METTL14, and Wilms Tumor 1‐Associating Protein (WTAP) were highly expressed in myeloid leukemia (Jaffrey & Kharas, 2017). These pioneering and encouraging discoveries require further investigation to discover the mechanistic details underlying the observed phenotypes.

## 2 THE SPECTRUM OF mRNA METHYL MARKS

**Figure 1:**
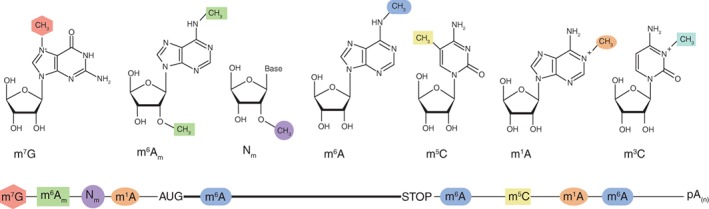
A schematic view of the structure and localization of methylated nucleosides in eukaryotic mRNA. The bold line represents the coding sequence, and the thin lines are 5′ and 3′ untranslated regions (UTRs). Abbreviations: m^7^G, 7‐methylguanosine; m^6^A_m_, N^6^,2′‐O‐methyaldenosine; Nm, 2′‐O‐ribose methylation; m^1^A, N^1^‐methyladenosine; m^6^A, N^6^‐methyladenosine; m^5^C, 5‐methylcytosine; m^3^C, 3‐methylcytosine

**Figure 2:**
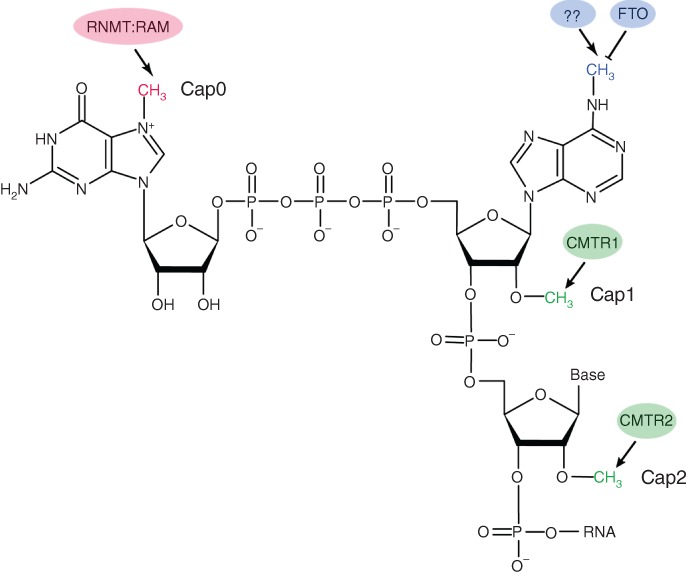
The chemical formula of the eukaryotic mRNA 5′‐m^7^G cap structure with the two downstream nucleotides included. Red: stable methyl group that forms the “Cap 0” structure (m^7^GpppN). Green: stable methyl groups that form the “Cap 1” (m^7^GpppNm) and “Cap 2” (m^7^GpppNmNm) structures. Blue: if the first nucleotide of the mRNA is adenosine, it can be further methylated at the N^6^ position of the base (m^7^Gpppm^6^A_m_). The enzymes responsible for methylation/demethylation of the specific groups are depicted

The spectrum of mRNA methyl marks includes, so far, six different modifications. RNA methylation may occur at the N1 and N6 atoms in adenosine, N3 and C5 in cytidine, N7 in guanosine, and at the 2′‐OH of ribose (Figure 1).

m^7^G, at the 5′ end of mRNA, marks the beginning of nearly all cellular mRNA transcribed by RNA polymerase II (RNAPII) (C. M. Wei et al., 1975b). In higher eukaryotes, the two nucleotides immediately adjacent to the cap show a complex methylation pattern (Figure 2). The characteristics and roles of the 2′‐O‐methylation (Nm) and N^6^,2′‐O‐methyaldenosine (m^6^A_m_) found next to the cap will be discussed later in this review. Most modifications found in mRNA, such as m^1^A, N^3^‐methylcytosine (m^3^C), N^5^‐methylcytosine (m^5^C), and m^6^A, were first observed in noncoding RNA (ncRNA), mainly in the abundant and stable transfer RNA (tRNA) and ribosomal RNA (rRNA).

Both adenosine modifications, m^1^A and m^6^A, were identified by the 1970s; however, more detailed molecular and biochemical studies began only recently. Both modifications are reversible, meaning that they can be added and subsequently erased from mRNA by specific enzymes.

m^1^A was first identified on rRNA and tRNA, where it is important for the formation of tertiary structure conformations (Helm, Giege, & Florentz, 1999), and in 2016 it was also reported in mRNA (Dominissini et al., 2016). It is written by TRMT6/TRM61A tRNA methyltransferase (X. Li et al., 2017; Safra et al., 2017), and it can be erased from tRNA and mRNA by the DNA/RNA demethylase ALKBH3 (Aas et al., 2003; Li, Xiong, Wang, et al., 2016; Ougland et al., 2004; Sundheim et al., 2006). In 2016 and 2017, several groups employed a method for site‐specific detection of m^1^A in mRNA, but different methodological approaches and data analyses led to controversial findings and conclusions. By using a combination of Dimroth rearrangement, antibody enrichment, and stalled reverse transcription, m^1^A was located at the 5′ untranslated region (UTR) and around start codons of several hundreds to several thousands of different mRNA transcripts (Dominissini et al., 2016; Li, Xiong, Wang, et al., 2016). These studies also revealed that m^1^A was dynamically regulated by diverse stress conditions, such as starvation or heat shock (Li, Xiong, Wang, et al., 2016), and its 5′ location was proposed to positively regulate translation initiation (Dominissini et al., 2016). However, by using reverse transcription‐based misincorporation and truncation at modified sites as well as more strict bioinformatics analysis, Safra et al. (2017) found m^1^A only in a handful of cytosolic mRNAs and a few mitochondrial mRNAs (in strong stem‐loop structures) and linked m^1^A to translational repression and developmental regulation. Since this field is very young, it needs further biochemical and chemical validations to accompany the high‐throughput sequencing studies. For instance, it is important to test the relevance of 5′ terminal m^1^As as well as to study the molecular mechanism that underlies the role of m^1^A at structured elements. Notably, m^1^A has been detected on *MALAT1*‐associated small cytoplasmic RNA (mascRNA), which is a small tRNA‐like RNA processed from *MALAT1* long noncoding RNA (lncRNA) by an unusual mechanism that involves RNase P and RNase Z (Wilusz, Freier, & Spector, 2008). It is possible that m^1^A, like in some tRNA, stabilizes the tertiary conformation of mascRNA and thus promotes its yet unknown function in the cytoplasm.

The additional methylation modifications m^3^C and m^5^C, which are also typically found in ncRNA (such as tRNA and rRNA) were recently also reported in mRNA (Clark, Evans, Dominissini, Zheng, & Pan, 2016; Cozen et al., 2015; Iwanami & Brown, 1968a, 1968b; Squires et al., 2012; Xu et al., 2017).

Two different enzymes are responsible for m^3^C deposition in tRNA, METTL2, and METTL6 (Xu et al., 2017), whereas METTL8 modifies mRNA (Xu et al., 2017). The presence of m^3^C on mRNA was indicated by high performance liquid chromatography coupled to mass spectrometry, but the specific locations are not yet known (Xu et al., 2017). Neither erasers nor readers have been identified for m^3^C so far.

Regarding m^5^C, its presence in mRNA remains debated. Several methods based on indirect m^5^C detection have been used to localize m^5^C in coding and ncRNA. These methods include m^5^C‐RNA immunoprecipitation (RIP), 5‐azacytidine‐mediated RIP (Aza‐IP), and methylation at individual nucleotide resolution crosslinking and immunoprecipitation (miCLIP) (Hussain, Aleksic, Blanco, Dietmann, & Frye, 2013; Li, Xiong, & Yi, 2016). For direct mapping of m^5^C in native RNA, bisulfite sequencing, a technique widely used to study DNA methylation, was adopted (Amort et al., 2017; David et al., 2017; Khoddami & Cairns, 2013; Legrand et al., 2017; Squires et al., 2012). Several of these studies reported thousands of m^5^C positions in poly(A) + RNA, with even higher m^5^C levels in nuclear fractions (Amort et al., 2017; Khoddami & Cairns, 2013; Squires et al., 2012). Thousands of m^5^C marks have been reported in plants. In *Arabidopsis*, the tRNA m^5^C methyltransferase TRM4B was linked to the methylation of thousands of sites in mRNA coding sequences, and m^5^C was suggested to play a role in mRNA stability and root development (David et al., 2017). The presence of m^5^C in eukaryotic mRNA has, however, been strongly questioned by the work of Legrand et al. (2017), who developed a stringent and statistically robust pipeline for whole‐transcriptome bisulfite sequencing data analysis and performed a comprehensive methylation analysis of mouse coding and ncRNA. Whereas their results revealed highly reproducible and robust detection of m^5^C in tRNA and rRNA, they did not show any significant m^5^C in mRNA (Legrand et al., 2017). However, some studies have linked m^5^C in mRNA and ncRNA with specific functions in mammals. For instance, the human mRNA export adaptor ALYREF was proposed to act as an m^5^C mRNA reader to regulate mRNA export from the nucleus (X. Yang et al., 2017), and loss of m^5^C methylation on vault ncRNAs caused aberrant processing into Argonaute‐associated small fragments that can function as microRNAs (miRNAs) (Hussain et al., 2013). Together, the presence and function of m^5^C in mammalian mRNA remains controversial.

Apart from simple methyl groups, more complex modifications can be found on RNA. Recently, the same family of enzymes that oxidize 5‐methylcytosine in DNA was reported to catalyze the formation of 5‐hydroxymethylcytosine in mammalian total RNA (5hmC) (Fu et al., 2014; Kohli & Zhang, 2013). In flies, a transcriptome wide study using an adapted version of methylation RNA immunoprecipitation sequencing (MeRIP‐seq) with 5hmrC antibodies, reported the presence of 5hmrC in many mRNA coding sequences, with particularly high levels in the brain (Delatte et al., 2016). They found that active translation is associated with high 5hmrC levels, and flies lacking the ten‐eleven translocation (TET) enzyme responsible for 5hmrC deposition have impaired brain development. In addition, the removal of m^6^A by the demethylase fat mass and obesity‐associated protein (FTO) is a multistep process that generates two intermediates, N^6^‐hydroxymethyladenosine (hm^6^A) and N^6^‐formyladenosine (f^6^A), with expected lifetimes of approximately 3 hr (Fu et al., 2013). It would be interesting to test whether these intermediates could also be recognized by different factors and change the fate of the modified transcripts.

## 3 THE MAMMALIAN m ^6^A EPITRANSCRIPTOME

The most abundant and extensively studied internal modification of mammalian mRNA is m^6^A. A growing body of work provides evidence about its role in nuclear pre‐mRNA processing. m^6^A was initially identified in the 1970s in both mammalian and viral RNA as a part of the cap structure at the first nucleotide downstream of m^7^G (Adams & Cory, 1975; Desrosiers et al., 1974; Dubin & Taylor, 1975). Thirty years later, two groups independently developed MeRIP‐Seq (or m^6^A‐Seq), a technique that provided the first data on m^6^A distribution within (pre‐)mRNA molecules (Dominissini et al., 2012; Meyer et al., 2012). This approach combines immunoprecipitation of fragmented RNA using an m^6^A‐specific antibody with high‐throughput sequencing and has been previously reviewed (Helm & Motorin, 2017; Li, Xiong, & Yi, 2016). These transcriptome‐wide m^6^A maps revealed that, in mammals, approximately 7,000 transcripts contain at least one m^6^A site. m^6^A marks were enriched in the proximity of the transcription start sites (TSS), around the stop codons, and at the beginning of the last exons (Dominissini et al., 2012; Meyer et al., 2012). Notably, the high‐throughput profiling results were highly consistent with previous biochemical analyses (Dubin & Taylor, 1975; Perry & Kelley, 1974; Wei & Moss, 1975). Apart from mRNA, m^6^A sites were also identified in various ncRNA, such as lncRNA (N. Liu et al., 2013; Meyer et al., 2012; Patil et al., 2016; Warda et al., 2017), primary‐microRNA (pri‐miRNA) (Alarcon, Lee, Goodarzi, Halberg, & Tavazoie, 2015), rRNA (Iwanami & Brown, 1968b), small nuclear RNA (snRNA) (Bringmann & Luhrmann, 1987), and tRNA (Saneyoshi, Harada, & Nishimura, 1969). The majority, but not all, of the internal mRNA m^6^A is found within a semi‐defined motif, namely RRACH (R = A/G, H = A/C/T) (Dominissini et al., 2012; Harper, Miceli, Roberts, & Manley, 1990; Meyer et al., 2012; Wei & Moss, 1977). Interestingly, at the 5′ UTR, the most prevalent motif is BCA (B = C/U/G), rather than RRACH (Linder et al., 2015). These methylated positions near the TSS are probably m^6^A_m_ sites, which contain an additional methyl group on the ribose (Mauer et al., 2017; C. Wei, Gershowitz, & Moss, 1975a). Another unique feature of these TSS‐proximal sites is that they are independent of WTAP, one of the main auxiliary proteins in the METTL3/14 complex (Schwartz et al., 2014). Overall, the RRACH motif appears to be specifically preferred by the methyltransferase METTL3 and the m^6^A_m_ sites at the TSS might be deposited by a different, as yet unknown methyltransferase.

### 3.1 Regulatory factors of the mammalian m^6^A epitranscriptome

#### 3.1.1 Enzymes that catalyze m^6^A modification: m^6^A writers

The N^6^‐methyl residues are deposited on adenosines by S‐adenosyl‐methionine (SAM)‐dependent methyltransferases. Mammals express at least two enzymes that have the potential to introduce m^6^A in mRNA, the heterodimer METTL3/METTL14 (METTL3/14) and METTL16. METTL3/14 (Liu et al., 2014) can associate with several adaptor proteins: WTAP (Liu et al., 2014; Ping et al., 2014; Schwartz et al., 2014), RBM15/15b (Patil et al., 2016), KIAA1429/VIRMA (Schwartz et al., 2014; Yue et al., 2018), HAKAI (Ruzicka et al., 2017; Yue et al., 2018) and ZC3H13 (Knuckles et al., 2018; Yue et al., 2018) (Table 1). However, the core of the active complex is formed by METTL3 and METTL14, homologous proteins that share 43% amino acid identity. They form a stable heterodimer, but only METTL3 contains catalytic methyltransferase activity, whereas METTL14 acts as a scaffold protein that promotes RNA interaction (Sledz & Jinek, 2016; Wang, Doxtader, & Nam, 2016; X. Wang, Feng, et al., 2016). This methylase complex is conserved across diverse eukaryotes, and homologs are found in all blasted metazoan genomes and many protozoa (our unpublished analyses).

**Table 1:** A summary of the known features of m^6^A protein factors, their molecular function, cellular localization, and a brief description of the m^6^A‐mediated role or activity of the mammalian m^6^A writers, erasers, and readers

Protein	Description	Localization	Molecular function	References
METTL3	Catalytic core of the METTL3/14 methyltransferase complex	N, C	Methyltransferase, transferase, binding RNA/protein/SAM	Liu et al. (2014), Sledz and Jinek (2016), X. Wang, Feng, et al. (2016)
MFTTL14	RNA‐binding scaffold of the METTL3/14 complex	N	Methyltransferase, transferase, binding RNA/protein/SAM	Liu et al. (2014), Sledz and Jinek (2016), X. Wang, Feng, et al. (2016)
WTAP	Adaptor protein of the MFTTL3/14 complex essential for localization to nuclear speckles	N	Binding protein	Ping et al. (2014), Schwartz et al. (2014)
VIRMA/KIAA1429	Adaptor protein of the METTL3/14 complex involved in guiding it to specific RNAs	N	Binding RNA	Schwartz et al. (2014), Yue et al. (2018)
RBM15/15B	Adaptor protein of the METTL3/14 complex involved in guiding it to specific RNAs	N	Binding protein/RNA/nucleic acid	Patil et al. (2016)
HAKAI	Adaptor protein of the MFTTL3/14 complex	N	Ubiquitin‐protein transferase/ligase activity, binding protein/metal ion	Ruzicka et al. (2017), Yue et al. (2018)
ZC3H13	Adaptor protein of the METTL3/14 complex required for protein protein interactions within the complex	N	Binding protein/RNA/metal ion	Yue et al. (2018), Knuckles et al. (2018)
METTL16	N6‐methyltransferase that methylates snRNAs and a subset of mRNAs	N	Methyltransferase, transferase, binding RNA/U6 snRNA 3′ end/RNA strem‐loop	Pendleton et al. (2017), Warda et al. (2017)
FTO	m^6^A/m^6^A_m_ RNA demethylase. Influences splicing and regulates cap‐independent translation after heat shock stress	N, C	Oxidoreductase activity, dioxygenase activity, oxidative RNA demethylase activity, oxidative DNA demethylase activity, binding metal ion	Jia et al. (2011), Bartosovic et al. (2017), Zhao et al. (2014)
ALKBH5	m^6^A RNA demethylase. Influences mRNA export	N	Oxidoreductase activity, dioxygenase activity, oxidative RNA demethylase activity, binding RNA/metal ion	Zheng et al. (2013)
YTHDF1	Direct m^6^A reader. Promotes translation of target m^6^A‐modified mRNAs	C	Binding RNA/m^6^A‐containing RNA/protein/ribosome	Wang et al. (2015)
YTHDF2	Direct m^6^A reader. Mediates decay and promotes cap‐independent translation after heat shock. YTHDF2‐mediated decay regulates mammalian oocyte rnaduration and zebrafish maternal mRNA clearance and haematopioetic stem cell specification	N, C	Binding RNA/m^6^A‐containing RNA/protein	X. Wang et al. (2014), Ivanova et al. (2017), Zhao et al. (2017), Zhou et al. (2015)
YTHDF3	Direct m^6^A reader. Promotes translation of target m6A‐modified mRNAs and circular RNAs	C	Binding RNA/m^6^A‐containing RNA/protein/ribosome	Y. Yang et al. (2017), A. Li et al. (2017)
YTHDC1	Direct m^6^A reader. Regulates splicing modulating the binding of SRSF3 and SRSF10. Mediates export of m6A‐modified mRNAs	N	Binding RNA/m^6^A containing RNA/protein	Xiao et al. (2016), Roundtree et al. (2017)
YTHDC2	Direct m^6^A reader. Regulates the meiotic program in mammalian germline	C	Binding RNA/m^6^A‐containing RNA/protein/ATP/RNA polymerase, helicase activity, ATP‐dependent RNA helicase activity, RNA‐dependent ATPase activity	Hsu et al. (2017), Wojtas et al. (2017)
HNRNPC	Indirect m^6^A reader. Mediates spiking events dependent on “m^6^A switch”	N	Binding RNA/m^6^A‐containing RNA/mRNA 3′UTR/ poly(U) RNA/telomerase RNA/nucleosomal DNA/RNA polymerase II proximal promoter and distal enhancer sequence‐specific DNA binding/protein	Liu et al. (2015)
HNRNPG	Indirect m^6^A reader. Mediates splicing events dependent on “m^6^A switch”	N	Binding RNA/protein	Liu et al. (2017)
HNRNPA2B1	Direct m^6^A reader. Promotes processing of m^6^A‐modified pri‐miRNA precursors. Regulates splicing of m^6^A‐modified transcripts	N, C	Binding RNA/ m^6^A containing RNA/mRNA 3′‐UTR/miRNA/pre‐mRNA intronic binding/single‐stranded telomeric DNA binding/G‐rich strand telomeric DNA binding/protein	Alarcon, Goodarzi, et al., 2015, Alarcon, Lee, et al., 2015
elF3	Direct m^6^A reader. Promotes translation of target mRNAs with m^6^A within the 5′ UTR	C	Binding RNA/protein, translation initiation factor activity	Meyer et al. (2015)

*Note*. N, nuclear; C, cytoplasmic localization.

Consistent with MeRIP‐seq experiments, RNA binding studies by photoactivatable ribonucleoside‐enhanced CLIP (PAR‐CLIP) revealed that METTL3 and METTL14 preferentially occupy regions that contain the RRACH motif (Liu et al., 2014; Ping et al., 2014). METTL3 and METTL14 colocalize in the nucleus, specifically in nuclear speckles (T. Chen et al., 2015; Liu et al., 2014; Ping et al., 2014); however, association with other regulatory proteins influences their cellular localization and RNA substrates (Patil et al., 2016; Ping et al., 2014; Schwartz et al., 2014). For example, interaction of METTL3 with *XIST* lncRNA is regulated by the RNA binding protein RBM15/15b (Patil et al., 2016). WTAP appears to mediate modification of the non‐TSS m^6^A sites (Schwartz et al., 2014), and it is essential for the nuclear localization of the complex. Downregulation of WTAP promotes METTL3/14 relocalization from nuclear speckles (Ping et al., 2014). The nuclear speckle localization of METTL3 was also disrupted upon knockdown of the miRNA processing factor Dicer (T. Chen, Hao, et al., 2015). Studies of polysome fractions from METTL3‐depleted cells suggested that METTL3 associates with polysomes and enhanced translation by recruiting eIF3 to the translation initiation complex (Lin, Choe, Du, Triboulet, & Gregory, 2016). However, the cytoplasmic role of METTL3 remains elusive and requires further clarification. Most of the studies indicate that METTL3/14 has primarily a nuclear function (Ke et al., 2017; Knuckles et al., 2017; Liu et al., 2014; Ping et al., 2014; Xiang et al., 2017).

The RRACH motif statistically occurs approximately once every 86 nucleotides, which is more than the experimentally observed methylation frequency. Despite intensive research in this field, it remains unclear how enrichment of m^6^A at specific locations is achieved. Taking into consideration the complexity of known interactors of METTL3 and METTL14 (Schwartz et al., 2014), it will be intriguing to see how other unknown mRNA binding factors influence m^6^A mRNA methylation.

The other m^6^A methyltransferase, METTL16, targets both ncRNA and protein coding mRNA (Pendleton et al., 2017; Warda et al., 2017). In contrast to METTL3/14, it does not associate with the RRACH motif. Therefore, some of the m^6^A localization studies based on this sequence motif may have underestimated the total number of sites (Warda et al., 2017). It is yet to be investigated whether METTL16 associates with any adaptor proteins. Additionally, thorough biochemical characterizations need to be performed to clarify its substrate specificity and enzymatic activities.

#### 3.1.2 The dynamics of m^6^A: m^6^A/m^6^A_m_ erasers

Similar to m^1^A (see above), m^6^A is a reversible mark. It can be readily removed in mammals by two currently known demethylases, FTO and ALKBH5 (Jia et al., 2011; Zheng et al., 2013) (Table 1). FTO and ALKBH5 are members of a protein family that harbors an ALKB domain. ALKB proteins are Fe^2+^/α‐ketoglutarate‐dependent dioxygenases that catalyze the conversion of methylated RNA and DNA nucleotides (Fedeles, Singh, Delaney, Li, & Essigmann, 2015). Similar to the methylase complex, both FTO and ALKBH5 are localized in nuclear speckles (Jia et al., 2011; Zheng et al., 2013). Although FTO can also be localized to the cytoplasm (Gulati et al., 2014), final m^6^A decoration, and thus the majority of demethylation, occurs on chromatin‐associated pre‐mRNA (Ke et al., 2017) to influence pre‐mRNA processing (Bartosovic et al., 2017; Zhao et al., 2014) (see below).

FTO substrate specificity has been debated numerous times. It was initially reported as a DNA demethylase that preferentially targeted 3‐methylthymine (3‐meT) in single‐stranded DNA (ssDNA) (Gerken et al., 2007). However, only a year later, in vitro studies demonstrated that recombinant human and mouse FTO displayed higher activity on 3‐methyluracil in ssRNA rather than 3‐meT in ssDNA (Jia et al., 2008). This view was again altered a few years later, when m^6^A RNA was identified as the main FTO substrate both in vitro and in vivo (Jia et al., 2011). Most recently, biochemical analyses combined with thin layer chromatography revealed dimethylated adenosine m^6^A_m_ as the major substrate of FTO in the cytoplasm (Mauer et al., 2017). The current view is that FTO preferentially targets m^6^A and m^6^A_m_; its activity on other modified RNA and DNA nucleotides needs to be clarified. Currently, ALKBH5 and FTO have only been found in vertebrates. Plants contain another m^6^A demethylase ALKBH9B (Martinez‐Perez et al., 2017).

#### 3.1.3 The recognition of m^6^A by specific reader proteins

m^6^A mRNA modification has emerged as a regulator of RNA structure (Liu et al., 2015; Spitale et al., 2015) and protein–RNA interactions. m^6^A functions are mediated by distinct RNA binding proteins termed m^6^A readers (Table 1). The m^6^A readers recognize modified RNAs either by direct recognition of m^6^A‐containing sequence or through afinity to particular RNA conformations that are sensitive to m^6^A.

m^6^A‐modifed RNA pulldown combined with mass spectrometry (MS) led to the identification of several m^6^A RNA binders (Dominissini et al., 2012). These proteins include several members of the YTH family (YT521, the founding member of the family), namely YTHDF1, YTHDF2, and YTHDF3 (Dominissini et al., 2012; Theler, Dominguez, Blatter, Boudet, & Allain, 2014; X. Wang, Lu, et al., 2014; Zhu et al., 2014). Although the YTH domain is found in several m^6^A reader proteins, direct m^6^A recognition does not fully rely on this domain. For instance, the translation initiation factor eIF3, which does not contain this domain, binds m^6^A in the 5′ UTR (Meyer et al., 2015). m^6^A can have both positive and negative effects on interactions with particular proteins. For instance, the binding affinity of ELAV‐1/HuR to some mRNA is reduced when they are m^6^A‐modified, although this reduction is largely dependent on the spatial distribution of m^6^A and HuR binding sites (X. Wang, Lu, et al., 2014).

Besides direct recognition, m^6^A can also indirectly affect protein binding by modulating adjacent binding sites that do not overlap with m^6^A. in vivo measurements of RNA secondary structures by in vivo click selective 2‐hydroxyl acylation and profiling experiment (icSHAPE) revealed decreased folding of methylated RNA sequences comparing to unmethylated; the finding underscored the role of m^6^A in regulating mRNA folding (Spitale et al., 2015). Disruption of dsRNA hairpins by m^6^A exposes ssRNA regions that facilitate their indirect recognition by ssRNA binding proteins. This m^6^A‐dependent RNA remodeling was termed “m^6^A switch” (Liu et al., 2015), and it can influence the binding of different RNA binding proteins (Liu et al., 2015, 2017). The versatility of m^6^A writer adaptors, the possibility for removal by erasers and a plethora of recognition mechanisms by readers (summarized in Table 1), confers m^6^A modification a regulatory potential that needs to be further studied.

## 4 THE ROLE OF METHYLATION IN PRE‐mRNA PROCESSING

Initial primary transcripts produced by RNAPII undergo several processing steps to become mature mRNA. For most pre‐mRNA, these steps include three main processes: addition and modification of a 5′ cap, removal of introns by RNA splicing, and formation of mature 3′ ends by cleavage and polyadenylation. Transcription and processing are not independent processes; they occur simultaneously and there is a crosstalk between the factors involved, largely through the carboxy‐terminal domain (CTD) of RNAPII (Lenasi & Barboric, 2013). Several lines of evidence support the notion that m^6^A deposition and removal occurs co‐transcriptionally (Baltz et al., 2012; Bartosovic et al., 2017; Ke et al., 2017; Knuckles et al., 2017; Slobodin et al., 2017). METTL3 can be co‐precipitated with slowed‐down RNAPII upon camptothecin (CPT) treatment (Slobodin et al., 2017), and METLL3 chromatin immunoprecipitation (ChIP) experiments demonstrated its direct association with chromatin (Knuckles et al., 2017). Furthermore, using a very different approach, Ke et al. (2017) reported that m^6^A deposition occurs during transcription while RNA is still attached to chromatin. The comparison of m^6^A‐CLIP profiles among chromatin‐associated RNA (pre‐mRNA), nucleoplasmic, and cytoplasmic mRNA did not reveal any significant differences; this finding implied that final m^6^A decoration is completed while the mRNA is still associated with the transcription locus. All the other m^6^A players, METTL16, FTO, and ALKBH5 target pre‐mRNA (Baltz et al., 2012; Bartosovic et al., 2017; Warda et al., 2017). However, their direct association with chromatin and transcription units remains unknown. Altogether, these studies suggest that m^6^A methylation/demethylation has the potential to regulate the very early steps of nuclear pre‐mRNA processing. Therefore, we will review the current knowledge on the role of m^6^A in pre‐mRNA processing and describe the open questions and discrepancies in the field.

### 4.1 The complex methyl code at the 5′ end of mRNA

Most eukaryotic mRNA is co‐transcriptionally modified at the 5′ end by a cap structure (Figure 2). The 5′ cap protects mRNA from 5′→3′ exoribonucleases, helps to recruit the molecular machinery necessary for translation initiation and splicing, and acts as an export signal for snRNA (Hocine, Singer, & Grunwald, 2010; Topisirovic, Svitkin, Sonenberg, & Shatkin, 2011).

The 5′ cap is formed by an unusual 5′‐5′ triphosphate linkage of a guanosine methylated at the seventh position of the guanosine ring (m^7^G) to the first 5′ terminal nucleotide of the mRNA (Wei, Gershowitz, & Moss, 1976; Wei & Moss, 1975). Guanosine m^7^G is the best characterized nucleotide methylation site of the cap (Ramanathan, Robb, & Chan, 2016; Topisirovic et al., 2011). However, 5′ mRNA termini have a more complex array of modifications. The two nucleotides immediately downstream of m^7^G can also be methylated at the second carbon of the ribose (2′‐OH position) to create 2′‐O‐methylation (Nm). If just the first nucleotide after m^7^G is methylated, it forms the so‐called cap 1 structure (m^7^GpppNm). In mammals, this methylation is performed by the enzyme CMTR1 (Belanger, Stepinski, Darzynkiewicz, & Pelletier, 2010). Additional 2′‐O‐methylation of the second position can occur in the cytoplasm, mediated by CMTR2 (Werner et al., 2011), to form cap 2 (m^7^GpppNmNm). These two additional methylations serve as a mark to distinguish self and nonself mRNA and to avoid the recognition of cellular mRNA by the innate immune system (Daffis et al., 2010; Zust et al., 2011). However, many viruses that replicate in the cytoplasm have evolved their own RNA‐modifying enzymes to mimic cellular mRNA in order to evade the host immune system (Daffis et al., 2010; Zust et al., 2011). 2′‐O‐methylation of viral mRNA avoids recognition by the cytoplasmic RNA sensor MDA5 and, consequently, the production of type I interferon, and thus subverts the host antiviral response (Zust et al., 2011).

In addition to ribose 2′‐O‐methylation, if the downstream adjacent nucleotide to m^7^G is A, it can be methylated at the sixth purine position to form m^6^A_m_ (C. Wei et al., 1975a). This methylation is reversible by FTO (Mauer et al., 2017) to produce 2′‐O‐methyaldenosine; however, it is unknown which enzyme adds the N^6^‐methyl group. These TSS‐associated m^6^A marks appear to play a major role in the cytoplasm to stabilize mRNA transcripts (Mauer et al., 2017) and activate translation initiation (Meyer et al., 2015; Y. Yang, Fan, et al., 2017; Zhou et al., 2015). The m^6^A marks within the 5′ UTR can act as so‐called m^6^A‐induced ribosome engagement sites (MIRES), and they promote mRNA translation under certain stress conditions (Meyer et al., 2015; Zhou et al., 2015). Based on the Zhou et al. (2015) study, heat shock induces relocalization of the cytoplasmic m^6^A reader YTHDF2 to the nucleus where it protects 5′ UTR m^6^As from demethylation by FTO. This event in turn promotes cap‐independent translation initiation by the direct recognition of m^6^A by eIF3, which recruits the pre‐initiation complex independently of the cap‐binding factor eIF4E (Meyer et al., 2015). A similar m^6^A‐driven translation initiation, but mediated by the m^6^A reader YTHDF3 and the translation initiation factor eIF4G2, was demonstrated with circular RNA (circRNA) (Y. Yang, Fan, et al., 2017).

In summary, the 5′ cap structure of mammalian mRNA can contain up to four methyl groups: the stable m^7^G, two Nm sites, and one reversible methylation site on transcripts that contain adenosine at the TSS (m^6^A_m_). It would be interesting to determine whether additional methyl groups are present on the cap and how the cellular machinery interprets this complex modification at the beginning of mRNA transcripts.

### 4.2 The role of RNA methylation in the regulation of pre‐mRNA splicing

m^6^A has been thought to play a critical role in constitutive splicing (Stoltzfus & Dane, 1982). The regulation of pre‐mRNA splicing is not fully understood. The spliceosome machinery is composed of small nuclear ribonucleoproteins (snRNPs), in which snRNA possess a number of nucleotide modifications, including base and 2′‐O‐ribose methylations. With some exceptions, the role and enzymatic pathways that produce most of these modifications are unknown. METTL16 catalyzes methylation at position A43 of U6 snRNA (Pendleton et al., 2017; Shimba, Bokar, Rottman, & Reddy, 1995; Warda et al., 2017). The exact function of this modification is unknown, but structural studies suggested that it might be important for local structure or base pairing in the U4/U6.U5 tri‐snRNP spliceosome (Agafonov et al., 2016).

**Figure 3:**
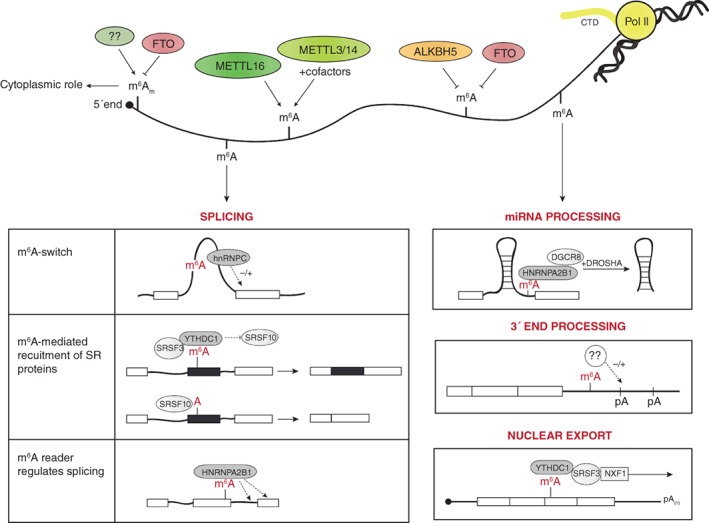
A schematic view of the role of the m^6^A methylation/demethylation pathway in nuclear pre‐mRNA processing. Writers, erasers, and known readers of m^6^A that play a role in splicing, 3′ end processing, and nuclear mRNA export are shown

Internal mRNA adenosine methylations were also linked to pre‐mRNA splicing in *cis*. Downregulation of m^6^A by METTL3 knockdown resulted in transcriptome‐wide alternative splicing (AS) changes (Dominissini et al., 2012), and a number of follow‐up studies have established robust links between both m^6^A and m^6^A factors and pre‐mRNA splicing (Bartosovic et al., 2017; Haussmann et al., 2016; Lence et al., 2016; Liu et al., 2015, 2017; Molinie et al., 2016; Patil et al., 2016; Xiao et al., 2016; Zhao et al., 2014). m^6^A modification may affect pre‐mRNA splicing by at least three different mechanisms (Figure 3), as described below:

1. m^6^A regulates the binding of specific splicing factors through RNA conformation. Mechanistically, m^6^A impacts the dynamics of RNA conformations that favor the transition from paired to unpaired RNA (Spitale et al., 2015). This mechanism was termed “m^6^A‐switch” and has the potential to regulate access of RNA binding proteins. RNA folding occurs co‐transcriptionally, and mRNA conformation immediately regulates co‐transcriptional processing, including splicing (Liu, Hu, & Zhang, 2016). To date, the presence of m^6^A was shown to facilitate the binding of hnRNPC to its target RNA and in turn influenced their splicing pattern (Liu et al., 2015). hnRNPC preferentially binds RNA on single‐stranded U‐tracts (>4 Us). Liu et al. (2017) demonstrated that the presence of m^6^A opposite to a U‐tract in a hairpin of *MALAT1* made the U‐tract more accessible and enhanced its interaction with hnRNPC.
2. Methylated mRNA recruits m^6^A reader proteins that bind in the vicinity of splice sites (SSs) and promote or repress recruitment of trans‐acting splicing factors. This mechanism occurred for YTHDC1, which bound m^6^A‐modified regions in the vicinity of the 5′ and 3′ SS and recruited the splicing factor SRSF3 while inhibiting the binding of SRSF10 (Xiao et al., 2016). SRSF3 and SRSF10 have opposing roles in AS; the former promotes exon inclusion and the latter exon skipping. Therefore, at exons with a SS that contains SRSF3 and 10 binding sites, the presence or absence of m^6^A, and in turn whether YTHDC1 is bound to that residue, can regulate AS (Xiao et al., 2016). YTHDC1 also plays a role in dosage compensation in mammals (Patil et al., 2016). m^6^A marks in the *X‐inactive specific transcript* (*XIST*) were required to mediate gene silencing of the X chromosome, although it is unknown how YTHDC1 binding contributes to gene silencing (Patil et al., 2016). Interestingly, the role of m^6^A in dosage compensation is conserved. In *Drosophila*, binding of the m^6^A reader YT521‐B to the methylated sex determination factor *Sex lethal* (*Sxl*), regulated the female‐specific AS of *sxl,* a key regulator of dosage compensation (Haussmann et al., 2016; Lence et al., 2016).
3. The third mechanism involves direct recognition of m^6^A‐modified motifs by splicing factors; only one example has been reported. The splicing factor hnRNPA2B1 was proposed to mediate some of the m^6^A‐METTL3‐dependent AS events, although the study lacks direct proof (Alarcon, Goodarzi, et al., 2015).

#### 4.2.1 The role of m^6^A demethylases in AS regulation

The role of m^6^A in AS regulation is supported by studies on m^6^A demethylases. FTO appears to regulate AS of a distinct subset of genes in mice and humans. In mouse adipocytes (3T3 cells), FTO‐mediated demethylation was proposed to affect splicing of a group of exons that rely on the splicing factor SRSF2 (Zhao et al., 2014). Among the genes, FTO seems important for exon exclusion in a transcript encoding the adipogenic regulatory factor *RUNX1T1*; this finding links FTO and adipogenesis (Merkestein et al., 2015; Zhao et al., 2014). In human HEK293T cells, FTO bound intronic regions in the proximity to alternatively spliced exons, and its activity affected both exon inclusion and exclusion for several hundred pre‐mRNAs (Bartosovic et al., 2017). The detailed mechanisms and splicing factor(s) involved have yet to be uncovered. ALKBH5 plays a key role in splicing regulation during spermatogenesis in mice (Tang et al., 2018). Its activity was required for correct splicing to generate long 3′ UTR transcripts in mitotic and meiotic male germ cells. These transcripts are crucial for timely degradation in later steps of spermatogenesis (Tang et al., 2018).

On the other hand, a study from the Darnel group suggested that the major role of m^6^A is mRNA stability rather than processing (Ke et al., 2017). They analyzed constitutive and AS in METTL3 knockout mouse embryonic stem cells (mESCs) and observed only minor changes compared with wild type cells. Nevertheless, alternatively spliced exons that contained m^6^A showed 4% and 1% more frequent inclusion and exclusion, respectively, in METTL3 knockout mESCs. In agreement with the proposed role of m^6^A in promoting mRNA degradation (Du et al., 2016; X. Wang, Lu, et al., 2014), they found that m^6^A correlated with rapid turnover (Ke et al., 2017).

AS is an extremely complex and highly regulated process that results in tissue and developmental stage‐specific splicing and proteome patterns (Nilsen & Graveley, 2010). Seemingly, m^6^A is one of the many AS regulators. It is important to further investigate which mechanisms and factors are involved in m^6^A‐regulated AS and also what regulates m^6^A deposition on distinct alternatively spliced transcripts.

### 4.3 Adenosine methylation in the formation of 3′ mRNA ends

Processing at the 3′ end of RNAPII transcripts involves a highly complex process and machinery capable of recognizing the precise localization of polyadenylation (poly(A)), endonucleolytic cleavage, and polyadenylation. It is mediated by a large multisubunit complex. Cleavage position is dictated by several sequence elements in the 3′ UTR. The best characterized example is the so‐called poly(A) site (PA). In humans, it is estimated that approximately 50% of protein coding genes contain more than one PA, so‐called alternative polyadenylation (APA) sites (Tian, Hu, Zhang, & Lutz, 2005). The differential usage of specific APAs subsequently determines the 3′ UTR length and presence or absence of additional regulatory sequences, such as miRNA binding sites, secondary structures, or motifs for RNA‐binding factors. The 3′ end quality thus impacts mRNA stability, localization, and translation rate.

With respect to the 3′ mRNA end, one of the two most prominent m^6^A sites is in the vicinity of stop codons and the beginning of the 3′ UTR (Batista et al., 2014; K. Chen et al., 2015; Dominissini et al., 2012; Linder et al., 2015; Meyer et al., 2012; Schwartz et al., 2014) or at the beginning of the last exon (Ke et al., 2015). Numerous studies established that m^6^A marks at 3′ mRNA ends regulate primarily cytoplasmic events such as stability and translation efficiency (X. Wang, Lu, et al., 2014; Wang et al., 2015; Y. Wang, Li, et al., 2014) (Box 2). These mechanisms have been studied in great detail (Fu, Dominissini, Rechavi, & He, 2014; Hoernes & Erlacher, 2017). However, several studies also provided evidence that m^6^A was involved in nuclear pre‐mRNA processing of 3′ ends (Bartosovic et al., 2017; Ke et al., 2015; Molinie et al., 2016).

BOX 2 **THE CYTOPLASMIC ROLE OF m^6^A**

m^6^A has been shown to regulate, in large part positively, mRNA translation and decay in the cytoplasm. The cytoplasmic reader YTHDF1 promoted translation of its target m^6^A‐modified mRNA (Wang et al., 2015). One of the most versatile m^6^A readers, YTHDF2, is connected with both translation and decay. Acting as a cytoplasmic reader, YTHDF2 promoted decay of m^6^A‐modified transcripts (X. Wang, Lu, et al., 2014) by recruiting the CCR4‐NOT complex (Du et al., 2016). This YTHDF2 role has been linked with clearance of specific mRNA transcripts during early development and oocyte maturation (Ivanova et al., 2017; Zhao et al., 2017) (see Box 1). On the other hand, as a nuclear reader, it is linked to translation. During heat shock, YTHDF2 protects m^6^A/m^6^A_m_ marks at the 5′ UTR of specific transcripts, namely *Hsp70*, from the demethylase FTO (Zhou et al., 2015). These methylated transcripts are recognized in the cytoplasm by eIF3 to promote cap‐independent translation (Meyer et al., 2015). Interestingly, m^6^A seems to play a dual role during heat shock. Under this condition, the m^6^A marks preferentially deposited by METTL3 in heat‐response transcripts also serve as marks for targeted and rapid degradation to ensure an acute response (Knuckles et al., 2017). Another protein of the same family, YTHDF3, promotes translation of target mRNA and circRNA (A. Li, Chen, et al., 2017; Y. Yang, Fan, et al., 2017). Only one study has linked methylation with increased stability. Transcripts with m^6^A_m_ at the 5′ cap seem to be more stable than monomethylated transcripts (m^7^GpppNm) probably due to their resistance to DCP2‐mediated decapping (Mauer et al., 2017).

Interestingly, the combination of m^6^A‐IP with UV‐crosslinking and high‐throughput sequencing uncovered that 70% of all m^6^A modifications were in the last exons, particularly in long last exons (Ke et al., 2015). It was proposed that long last exons could carry more m^6^A residues, and this increase in modified residues could inhibit proximal APA selection. However, downregulation of METTL3, METTL14, and WTAP resulted in differential APA selection in both proximal and distal sites in a subset of mRNAs (Ke et al., 2015). The study did not provide any further insights on the possible molecular mechanism of the m^6^A‐linked APA selection.

Since the development of the first transcriptome‐wide methods to study m^6^A location and distribution along mRNA, different approaches have improved several aspects of the technique (K. Chen, Lu, et al., 2015; Linder et al., 2015; Liu et al., 2013; Molinie et al., 2016). One improvement was the development of the so‐called “m^6^A‐LAIC‐Seq” (Molinie et al., 2016) that relies on sequencing intact full‐length transcripts from m^6^A enriched and nonenriched samples. Sequencing nonfragmented RNA allows comparison of m^6^A enrichment on transcript variants of individual genes. Using this novel technique, Molinie et al. (2016) noted that m^6^A‐enriched transcripts tend to more frequently use proximal APA sites than their corresponding nonmethylated partners. On the other hand, for the total RNA population (input), they observed that m^6^A levels positively correlated with 3′ UTR length, in accordance with the findings by Ke et al. that m^6^A accumulated in long last exons (Ke et al., 2015; Molinie et al., 2016). This apparent discrepancy could be explained by m^6^A‐mediated degradation. Molinie et al. proposed that these m^6^A‐enriched transcripts with short 3′ UTRs turn over more rapidly and therefore are underrepresented in the total RNA population. This model would also be supported by recent findings about FTO. Depletion of FTO by CRISPR‐Cas9 in HEK293 cells resulted in longer 3′ termini for a large subset of genes, which coincided with the position of validated APA sites (Bartosovic et al., 2017; Gruber et al., 2016). However, whether stabilization of FTO‐dependent m^6^A marks really promotes degradation of short 3′ UTR transcripts remains to be tested.

In summary, there are myriad questions and still very few answers regarding the potential role of m^6^A in 3′ end processing. It would be interesting and challenging to explore whether some of the players in the m^6^A pathway are involved with 3′ end processing machinery and help in poly(A) site selection (Figure 3).

### 4.4 RNA methylation as a regulator of mRNA export to the cytoplasm

Mechanisms that connect efficient mRNA processing and export are critical for proper gene expression. Previous observations suggested that the cytoplasmic appearance of mRNA is dependent on m^6^A methylation (Camper, Albers, Coward, & Rottman, 1984; Finkel & Groner, 1983). Reducing m^6^A levels using the S‐adenosylhomocysteine analog S‐tubercidinylhomocysteine (STH) in HeLa cells resulted in delayed nuclear export of mature mRNA (Camper et al., 1984), and the same effect was observed upon METTL3 knockdown (Fustin et al., 2013). Correspondingly, ALKBH5 depletion led to rapid cytoplasmic mRNA appearance (Zheng et al., 2013).

Mechanistically, this process is mediated, at least for some mRNA, by YTHDC1. YTHDC1 incorporates the methylated target mRNA into the nuclear export pathway by interaction with SRSF3 (Roundtree et al., 2017). SRSF3 then acts as an adaptor between YTHDC1 and nuclear export factor 1 (NXF1) to facilitate the export of the m^6^A‐modified mRNA bound by the reader protein (Roundtree et al., 2017; Figure 3).

These pioneer studies have proposed mechanistic insights on mRNA methylation‐driven nuclear export. Future studies will reveal the extent of this regulation and the molecular details which link methylation, pre‐mRNA processing, and export.

### 4.5 Methylation and small RNA processing

The accumulation of methyl marks, namely m^6^A at 3′ UTRs, partially correlated with miRNA binding sites (T. Chen, Hao, et al., 2015; Dominissini et al., 2012; Meyer et al., 2012). In addition, knockdown of Dicer, the endonuclease responsible for producing mature miRNA, reduced m^6^A levels in mammalian neuronal stem cells and HeLa cells and caused delocalization of METTL3 from nuclear speckles (T. Chen, Hao, et al., 2015). However, METTL3 and Dicer do not interact in vivo, and the changes in m^6^A levels seem to be mediated directly by miRNA; exogenous introduction of miRNA directed against nonmethylated transcripts increased m^6^A levels on their target sites (T. Chen, Hao, et al., 2015). It was proposed that miRNA could help to direct METTL3/14 to modify specific sites, although no further mechanistic details were provided (T. Chen, Hao, et al., 2015).

In turn, m^6^A marks were also detected on pri‐miRNA. m^6^A promoted pri‐miRNA processing by enhancing its recognition by DGCR8 (Alarcon, Lee, et al., 2015). This process was mediated by the nuclear m^6^A reader HNRNPA2B1, which recognized the methyl marks and interacted with the microprocessor complex and DGCR8 (Alarcon, Goodarzi, et al., 2015) (Figure 3). Altogether, these works suggest that METTL3 and miRNA processing pathways are co‐regulated and the interplay between them affects both mRNA methylation and miRNA processing.

## 5 CONCLUSION

In the early days of RNA research, it was already recognized that this molecule bears variable chemical modifications. However, due to technical limitations, the majority of research on RNA modifications was restricted to the three classes of highly expressed and stable RNA: rRNA, tRNA, and snRNA. Only recently have major technical advances revived interest in RNA biology and its processing, modification, and physiology. This expanding interest, in turn, acts as driving force to keep improving the technological tools. Biochemical approaches, coupled to high‐throughput sequencing, have made it possible to study not only whole transcriptomes but also to map individual modifications and reveal the function of RNA‐modifying enzymes or protein binding factors. All these tools would be inconceivable without increasing progress in computing power and development of bioinformatics tools. Improved sensitivity of mass spectrometry made it the gold standard for the identification of new chemical modifications and their assignment to specific types of RNA. The revolutionizing step in m^6^A studies was the development and utilization of an anti‐m^6^A antibody for immunoprecipitation of methylated RNA fragments followed by RNA sequencing. All of these factors, combined with developments in cell manipulation, including RNAi tools, CRISPR‐Cas9 gene editing, and protein tagging, allowed the identification of several writers, readers, and erasers in the field of epitranscriptomics.

A certain degree of controversy and inconsistency currently exists in the field. Careful steps must be taken to clarify the presence of methylation other than m^6^A in the eukaryotic mRNA. The biggest challenge to this task is still how to precisely and reliably detect individual modifications with single nucleotide resolution. Rectification will include improved protocols for pure mRNA isolation (free of contaminating ncRNA) and development of new approaches that allow direct, site‐specific detection of methyl marks without the use of indirect tools (e.g., antibody enrichment or relying on reverse transcriptase “errors”). Most importantly, further elaboration of robust and available bioinformatics analyses tools should be a key step in comparative analyses of data obtained from various groups. Currently, attempts are being made to interconnect the published data in databases (Boccaletto et al., 2018).

It will be crucial to understand the functions of methyl marks on mRNA and other ncRNA. mRNA exhibits a complex co‐ and post‐transcriptional life, which in the nucleus involves a number of processing and modification steps, stability, and nucleo‐cytoplasmic export. Most of these processes are interconnected. Several studies implied that methylation and demethylation occur co‐transcriptionally; therefore, it will be important to reveal to what extent methylation can affect transcription, and *vis‐a‐vis*, how transcription efficiency may regulate mRNA methylation patterns. The dynamics of m^6^A and m^1^A resembles the dynamics of DNA modifications, which play a crucial role in gene expression regulation. Since the modifiers are to a certain extent homologous to DNA‐modifying proteins, it will be interesting to address whether there is any crosstalk between epigenetic and epitranscriptomic pathways.

The expanding interest in the field has led to the identification and characterization of the key factors of the m^6^A mRNA pathway. The mammalian methyltransferase core complex has been characterized biochemically, structurally, and to certain extent biologically. However, we still do not know how auxiliary factors, such as Virilizer homolog, WTAP, or RBM15/15b, regulate methylation. One of the big questions that remains is what defines the final m^6^A (or other mark) pattern. m^6^A and its writers have been located to RRACH motifs, but only a very small portion of such motifs at specific mRNA locations are methylated. This phenomenon could be regulated by adaptor factors, including RNA binding proteins, METTL3/14 auxiliary factors, or even other RNAs (e.g., miRNA) (T. Chen, Hao, et al., 2015; Patil et al., 2016). Additionally, not much is known regarding the selectivity of readers, that is, whether different readers bind to distinct subsets of sites and how such specificity is achieved. Alternatively, the activity and selectivity of RNA demethylases could determine which marks can persist in a given RNA molecule. For m^6^A, at least two mRNA m^6^A methyltransferases and two demethylases have been uncovered. The level of target and functional redundancy between these factors is yet unknown. It is also unknown what enzyme(s) deposit(s) methyl groups on purine rings in the 5′ terminal m^6^A_m_. Based on a recent report from Meyer et al., FTO prefers m^6^A_m_ rather than m^6^A (Mauer et al., 2017). This finding needs to be further integrated with other published results that link FTO nuclear function to m^6^A.

Taken together, a number of studies have increased significantly our awareness about the importance of methyl marks on coding RNA and have given rise to the new field of mRNA epitranscriptome. The field is still in its infancy, and there are exciting opportunities for new fundamental discoveries.

## CONFLICT OF INTEREST

The authors have declared no conflicts of interest for this article.

## RELATED WIREs ARTICLES

https://doi.org/10.1002/wrna.1166

https://doi.org/10.1002/wrna.79

https://doi.org/10.1002/wrna.1380

https://doi.org/10.1002/wrna.1450

## References

[wrna1489-bib-0001] Aas, P. A. , Otterlei, M. , Falnes, P. O. , Vagbo, C. B. , Skorpen, F. , Akbari, M. , … Krokan, H. E. (2003). Human and bacterial oxidative demethylases repair alkylation damage in both RNA and DNA. Nature, 421(6925), 859–863. 10.1038/nature01363 12594517

[wrna1489-bib-0002] Adams, J. M. , & Cory, S. (1975). Modified nucleosides and bizarre 5′‐termini in mouse myeloma mRNA. Nature, 255(5503), 28–33. 1128665 10.1038/255028a0

[wrna1489-bib-0003] Agafonov, D. E. , Kastner, B. , Dybkov, O. , Hofele, R. V. , Liu, W. T. , Urlaub, H. , … Stark, H. (2016). Molecular architecture of the human U4/U6.U5 tri‐snRNP. Science, 351(6280), 1416–1420. 10.1126/science.aad2085 26912367

[wrna1489-bib-0004] Alarcon, C. R. , Goodarzi, H. , Lee, H. , Liu, X. , Tavazoie, S. , & Tavazoie, S. F. (2015). HNRNPA2B1 is a mediator of m(6)A‐dependent nuclear RNA processing events. Cell, 162(6), 1299–1308. 10.1016/j.cell.2015.08.011 26321680 PMC4673968

[wrna1489-bib-0005] Alarcon, C. R. , Lee, H. , Goodarzi, H. , Halberg, N. , & Tavazoie, S. F. (2015). N6‐methyladenosine marks primary microRNAs for processing. Nature, 519(7544), 482–485. 10.1038/nature14281 25799998 PMC4475635

[wrna1489-bib-0006] Amort, T. , Rieder, D. , Wille, A. , Khokhlova‐Cubberley, D. , Riml, C. , Trixl, L. , … Lusser, A. (2017). Distinct 5‐methylcytosine profiles in poly(A) RNA from mouse embryonic stem cells and brain. Genome Biology, 18(1), 1 10.1186/s13059-016-1139-1 28077169 PMC5225599

[wrna1489-bib-0007] Baltz, A. G. , Munschauer, M. , Schwanhausser, B. , Vasile, A. , Murakawa, Y. , Schueler, M. , … Landthaler, M. (2012). The mRNA‐bound proteome and its global occupancy profile on protein‐coding transcripts. Molecular Cell, 46(5), 674–690. 10.1016/j.molcel.2012.05.021 22681889

[wrna1489-bib-0008] Bartosovic, M. , Molares, H. C. , Gregorova, P. , Hrossova, D. , Kudla, G. , & Vanacova, S. (2017). N6‐methyladenosine demethylase FTO targets pre‐mRNAs and regulates alternative splicing and 3′‐end processing. Nucleic Acids Research. 10.1093/nar/gkx778 PMC5737695 28977517

[wrna1489-bib-0009] Batista, P. J. , Molinie, B. , Wang, J. , Qu, K. , Zhang, J. , Li, L. , … Chang, H. Y. (2014). m(6)A RNA modification controls cell fate transition in mammalian embryonic stem cells. Cell Stem Cell, 15(6), 707–719. 10.1016/j.stem.2014.09.019 25456834 PMC4278749

[wrna1489-bib-0010] Belanger, F. , Stepinski, J. , Darzynkiewicz, E. , & Pelletier, J. (2010). Characterization of hMTr1, a human Cap1 2′‐O‐ribose methyltransferase. Journal of Biological Chemistry, 285(43), 33037–33044. 10.1074/jbc.M110.155283 20713356 PMC2963352

[wrna1489-bib-0011] Boccaletto, P. , Machnicka, M. A. , Purta, E. , Piatkowski, P. , Baginski, B. , Wirecki, T. K. , … Bujnicki, J. M. (2018). MODOMICS: A database of RNA modification pathways. 2017 update. Nucleic Acids Research, 46(D1), D303–D307. 10.1093/nar/gkx1030 29106616 PMC5753262

[wrna1489-bib-0012] Bringmann, P. , & Luhrmann, R. (1987). Antibodies specific for N6‐methyladenosine react with intact snRNPs U2 and U4/U6. FEBS Letters, 213(2), 309–315. 2951275 10.1016/0014-5793(87)81512-0

[wrna1489-bib-0013] Camper, S. A. , Albers, R. J. , Coward, J. K. , & Rottman, F. M. (1984). Effect of undermethylation on mRNA cytoplasmic appearance and half‐life. Molecular and Cellular Biology, 4(3), 538–543. 6201720 10.1128/mcb.4.3.538-543.1984 PMC368733

[wrna1489-bib-0014] Chen, K. , Lu, Z. , Wang, X. , Fu, Y. , Luo, G. Z. , Liu, N. , … He, C. (2015). High‐resolution N(6)‐methyladenosine (m(6)A) map using photo‐crosslinking‐assisted m(6) A sequencing. Angewandte Chemie, 54(5), 1587–1590. 10.1002/anie.201410647 25491922 PMC4396828

[wrna1489-bib-0015] Chen, T. , Hao, Y. J. , Zhang, Y. , Li, M. M. , Wang, M. , Han, W. , … Zhou, Q. (2015). m(6)A RNA methylation is regulated by microRNAs and promotes reprogramming to pluripotency. Cell Stem Cell, 16(3), 289–301. 10.1016/j.stem.2015.01.016 25683224

[wrna1489-bib-0016] Clark, W. C. , Evans, M. E. , Dominissini, D. , Zheng, G. , & Pan, T. (2016). tRNA base methylation identification and quantification via high‐throughput sequencing. RNA, 22(11), 1771–1784. 10.1261/rna.056531.116 27613580 PMC5066629

[wrna1489-bib-0017] Cozen, A. E. , Quartley, E. , Holmes, A. D. , Hrabeta‐Robinson, E. , Phizicky, E. M. , & Lowe, T. M. (2015). ARM‐seq: AlkB‐facilitated RNA methylation sequencing reveals a complex landscape of modified tRNA fragments. Nature Methods, 12(9), 879–884. 10.1038/nmeth.3508 26237225 PMC4553111

[wrna1489-bib-0018] Cui, Q. , Shi, H. , Ye, P. , Li, L. , Qu, Q. , Sun, G. , … Shi, Y. (2017). m6A RNA methylation regulates the self‐renewal and tumorigenesis of glioblastoma stem cells. Cell Reports, 18(11), 2622–2634. 10.1016/j.celrep.2017.02.059 28297667 PMC5479356

[wrna1489-bib-0019] Daffis, S. , Szretter, K. J. , Schriewer, J. , Li, J. , Youn, S. , Errett, J. , … Diamond, M. S. (2010). 2′‐O methylation of the viral mRNA cap evades host restriction by IFIT family members. Nature, 468(7322), 452–456. 10.1038/nature09489 21085181 PMC3058805

[wrna1489-bib-0020] David, R. , Burgess, A. , Parker, B. , Li, J. , Pulsford, K. , Sibbritt, T. , … Searle, I. R. (2017). Transcriptome‐wide mapping of RNA 5‐methylcytosine in Arabidopsis mRNAs and noncoding RNAs. Plant Cell, 29(3), 445–460. 10.1105/tpc.16.00751 28062751 PMC5385953

[wrna1489-bib-0021] Delatte, B. , Wang, F. , Ngoc, L. V. , Collignon, E. , Bonvin, E. , Deplus, R. , … Fuks, F. (2016). RNA biochemistry. Transcriptome‐wide distribution and function of RNA hydroxymethylcytosine. Science, 351(6270), 282–285. 10.1126/science.aac5253 26816380

[wrna1489-bib-0022] Desrosiers, R. , Friderici, K. , & Rottman, F. (1974). Identification of methylated nucleosides in messenger RNA from Novikoff hepatoma cells. Proceedings of the National Academy of Sciences of the United States of America, 71(10), 3971–3975. 4372599 10.1073/pnas.71.10.3971 PMC434308

[wrna1489-bib-0023] Dominissini, D. , Moshitch‐Moshkovitz, S. , Schwartz, S. , Salmon‐Divon, M. , Ungar, L. , Osenberg, S. , … Rechavi, G. (2012). Topology of the human and mouse m6A RNA methylomes revealed by m6A‐seq. Nature, 485(7397), 201–206. 10.1038/nature11112 22575960

[wrna1489-bib-0024] Dominissini, D. , Nachtergaele, S. , Moshitch‐Moshkovitz, S. , Peer, E. , Kol, N. , Ben‐Haim, M. S. , … He, C. (2016). The dynamic N(1)‐methyladenosine methylome in eukaryotic messenger RNA. Nature, 530(7591), 441–446. 10.1038/nature16998 26863196 PMC4842015

[wrna1489-bib-0025] Du, H. , Zhao, Y. , He, J. , Zhang, Y. , Xi, H. , Liu, M. , … Wu, L. (2016). YTHDF2 destabilizes m(6)A‐containing RNA through direct recruitment of the CCR4‐NOT deadenylase complex. Nature Communications, 7, 12626 10.1038/ncomms12626 PMC5007331 27558897

[wrna1489-bib-0026] Dubin, D. T. , & Taylor, R. H. (1975). The methylation state of poly A‐containing messenger RNA from cultured hamster cells. Nucleic Acids Research, 2(10), 1653–1668. 1187339 10.1093/nar/2.10.1653 PMC343535

[wrna1489-bib-0027] Edmonds, M. , Vaughan, M. H., Jr. , & Nakazato, H. (1971). Polyadenylic acid sequences in the heterogeneous nuclear RNA and rapidly‐labeled polyribosomal RNA of HeLa cells: Possible evidence for a precursor relationship. Proceedings of the National Academy of Sciences of the United States of America, 68(6), 1336–1340. 5288383 10.1073/pnas.68.6.1336 PMC389184

[wrna1489-bib-0028] Fedeles, B. I. , Singh, V. , Delaney, J. C. , Li, D. , & Essigmann, J. M. (2015). The AlkB family of Fe(II)/alpha‐ketoglutarate‐dependent dioxygenases: Repairing nucleic acid alkylation damage and beyond. Journal of Biological Chemistry, 290(34), 20734–20742. 10.1074/jbc.R115.656462 26152727 PMC4543635

[wrna1489-bib-0029] Finkel, D. , & Groner, Y. (1983). Methylations of adenosine residues (m6A) in pre‐mRNA are important for formation of late simian virus 40 mRNAs. Virology, 131(2), 409–425. 6318439 10.1016/0042-6822(83)90508-1

[wrna1489-bib-0030] Fu, L. , Guerrero, C. R. , Zhong, N. , Amato, N. J. , Liu, Y. , Liu, S. , … Wang, Y. (2014). Tet‐mediated formation of 5‐hydroxymethylcytosine in RNA. Journal of the American Chemical Society, 136(33), 11582–11585. 10.1021/ja505305z 25073028 PMC4140497

[wrna1489-bib-0031] Fu, Y. , Dominissini, D. , Rechavi, G. , & He, C. (2014). Gene expression regulation mediated through reversible m(6)A RNA methylation. Nature Reviews. Genetics, 15(5), 293–306. 10.1038/nrg3724 24662220

[wrna1489-bib-0032] Fu, Y. , Jia, G. , Pang, X. , Wang, R. N. , Wang, X. , Li, C. J. , … He, C. (2013). FTO‐mediated formation of N6‐hydroxymethyladenosine and N6‐formyladenosine in mammalian RNA. Nature Communications, 4, 1798 10.1038/ncomms2822 PMC3658177 23653210

[wrna1489-bib-0033] Fustin, J. M. , Doi, M. , Yamaguchi, Y. , Hida, H. , Nishimura, S. , Yoshida, M. , … Okamura, H. (2013). RNA‐methylation‐dependent RNA processing controls the speed of the circadian clock. Cell, 155(4), 793–806. 10.1016/j.cell.2013.10.026 24209618

[wrna1489-bib-0034] Gerken, T. , Girard, C. A. , Tung, Y. C. , Webby, C. J. , Saudek, V. , Hewitson, K. S. , … Schofield, C. J. (2007). The obesity‐associated FTO gene encodes a 2‐oxoglutarate‐dependent nucleic acid demethylase. Science, 318(5855), 1469–1472. 10.1126/science.1151710 17991826 PMC2668859

[wrna1489-bib-0035] Geula, S. , Moshitch‐Moshkovitz, S. , Dominissini, D. , Mansour, A. A. , Kol, N. , Salmon‐Divon, M. , … Hanna, J. H. (2015). Stem cells. m6A mRNA methylation facilitates resolution of naive pluripotency toward differentiation. Science, 347(6225), 1002–1006. 10.1126/science.1261417 25569111

[wrna1489-bib-0036] Gruber, A. J. , Schmidt, R. , Gruber, A. R. , Martin, G. , Ghosh, S. , Belmadani, M. , … Zavolan, M. (2016). A comprehensive analysis of 3′ end sequencing data sets reveals novel polyadenylation signals and the repressive role of heterogeneous ribonucleoprotein C on cleavage and polyadenylation. Genome Research, 26(8), 1145–1159. 10.1101/gr.202432.115 27382025 PMC4971764

[wrna1489-bib-0037] Gulati, P. , Avezov, E. , Ma, M. , Antrobus, R. , Lehner, P. , O'Rahilly, S. , & Yeo, G. S. (2014). Fat mass and obesity‐related (FTO) shuttles between the nucleus and cytoplasm. Bioscience Reports, 34(5), 621–628. 10.1042/BSR20140111 PMC4206862 25242086

[wrna1489-bib-0038] Harper, J. E. , Miceli, S. M. , Roberts, R. J. , & Manley, J. L. (1990). Sequence specificity of the human mRNA N6‐adenosine methylase in vitro. Nucleic Acids Research, 18(19), 5735–5741. 2216767 10.1093/nar/18.19.5735 PMC332308

[wrna1489-bib-0039] Haussmann, I. U. , Bodi, Z. , Sanchez‐Moran, E. , Mongan, N. P. , Archer, N. , Fray, R. G. , & Soller, M. (2016). m6A potentiates Sxl alternative pre‐mRNA splicing for robust *Drosophila* sex determination. Nature, 540(7632), 301–304. 10.1038/nature20577 27919081

[wrna1489-bib-0040] Helm, M. , Giege, R. , & Florentz, C. (1999). A Watson‐Crick base‐pair‐disrupting methyl group (m1A9) is sufficient for cloverleaf folding of human mitochondrial tRNALys. Biochemistry, 38(40), 13338–13346. 10529209 10.1021/bi991061g

[wrna1489-bib-0041] Helm, M. , & Motorin, Y. (2017). Detecting RNA modifications in the epitranscriptome: Predict and validate. Nature Reviews. Genetics, 18(5), 275–291. 10.1038/nrg.2016.169 28216634

[wrna1489-bib-0042] Hocine, S. , Singer, R. H. , & Grunwald, D. (2010). RNA processing and export. Cold Spring Harbor Perspectives in Biology, 2(12), a000752 10.1101/cshperspect.a000752 20961978 PMC2982171

[wrna1489-bib-0043] Hoernes, T. P. , & Erlacher, M. D. (2017). Translating the epitranscriptome. WIREs RNA, 8(1), e1375 10.1002/wrna.1375 PMC5215311 27345446

[wrna1489-bib-0044] Hsu, P. J. , Zhu, Y. , Ma, H. , Guo, Y. , Shi, X. , Liu, Y. , … He, C. (2017). Ythdc2 is an N6‐methyladenosine binding protein that regulates mammalian spermatogenesis. Cell Research, 27(9), 1115–1127. 10.1038/cr.2017.99 28809393 PMC5587856

[wrna1489-bib-0045] Hussain, S. , Aleksic, J. , Blanco, S. , Dietmann, S. , & Frye, M. (2013). Characterizing 5‐methylcytosine in the mammalian epitranscriptome. Genome Biology, 14(11), 215 10.1186/gb4143 24286375 PMC4053770

[wrna1489-bib-0046] Hussain, S. , Sajini, A. A. , Blanco, S. , Dietmann, S. , Lombard, P. , Sugimoto, Y. , … Frye, M. (2013). NSun2‐mediated cytosine‐5 methylation of vault noncoding RNA determines its processing into regulatory small RNAs. Cell Reports, 4(2), 255–261. 10.1016/j.celrep.2013.06.029 23871666 PMC3730056

[wrna1489-bib-0047] Ivanova, I. , Much, C. , Di Giacomo, M. , Azzi, C. , Morgan, M. , Moreira, P. N. , … O'Carroll, D. (2017). The RNA m6A reader YTHDF2 is essential for the post‐transcriptional regulation of the maternal transcriptome and oocyte competence. Molecular Cell, 67(6), 1059, e1054–1067. 10.1016/j.molcel.2017.08.003 PMC5613143 28867294

[wrna1489-bib-0048] Iwanami, Y. , & Brown, G. M. (1968a). Methylated bases of ribosomal ribonucleic acid from HeLa cells. Archives of Biochemistry and Biophysics, 126(1), 8–15. 5671075 10.1016/0003-9861(68)90553-5

[wrna1489-bib-0049] Iwanami, Y. , & Brown, G. M. (1968b). Methylated bases of transfer ribonucleic acid from HeLa and L cells. Archives of Biochemistry and Biophysics, 124(1), 472–482. 5661617 10.1016/0003-9861(68)90355-x

[wrna1489-bib-0050] Jaffrey, S. R. , & Kharas, M. G. (2017). Emerging links between m6A and misregulated mRNA methylation in cancer. Genome Medicine, 9(1), 2 10.1186/s13073-016-0395-8 28081722 PMC5228104

[wrna1489-bib-0051] Jia, G. , Fu, Y. , Zhao, X. , Dai, Q. , Zheng, G. , Yang, Y. , … He, C. (2011). N6‐methyladenosine in nuclear RNA is a major substrate of the obesity‐associated FTO. Nature Chemical Biology, 7(12), 885–887. 10.1038/nchembio.687 22002720 PMC3218240

[wrna1489-bib-0052] Jia, G. , Yang, C. G. , Yang, S. , Jian, X. , Yi, C. , Zhou, Z. , & He, C. (2008). Oxidative demethylation of 3‐methylthymine and 3‐methyluracil in single‐stranded DNA and RNA by mouse and human FTO. FEBS Letters, 582(23–24), 3313–3319. 10.1016/j.febslet.2008.08.019 18775698 PMC2577709

[wrna1489-bib-0053] Ke, S. , Alemu, E. A. , Mertens, C. , Gantman, E. C. , Fak, J. J. , Mele, A. , … Darnell, R. B. (2015). A majority of m6A residues are in the last exons, allowing the potential for 3′ UTR regulation. Genes & Development, 29(19), 2037–2053. 10.1101/gad.269415.115 26404942 PMC4604345

[wrna1489-bib-0054] Ke, S. , Pandya‐Jones, A. , Saito, Y. , Fak, J. J. , Vagbo, C. B. , Geula, S. , … Darnell, R. B. (2017). m6A mRNA modifications are deposited in nascent pre‐mRNA and are not required for splicing but do specify cytoplasmic turnover. Genes & Development, 31(10), 990–1006. 10.1101/gad.301036.117 28637692 PMC5495127

[wrna1489-bib-0055] Khoddami, V. , & Cairns, B. R. (2013). Identification of direct targets and modified bases of RNA cytosine methyltransferases. Nature Biotechnology, 31(5), 458–464. 10.1038/nbt.2566 PMC3791587 23604283

[wrna1489-bib-0056] Knuckles, P. , Carl, S. H. , Musheev, M. , Niehrs, C. , Wenger, A. , & Buhler, M. (2017). RNA fate determination through cotranscriptional adenosine methylation and microprocessor binding. Nature Structural & Molecular Biology, 24(7), 561–569. 10.1038/nsmb.3419 28581511

[wrna1489-bib-0057] Knuckles, P. , Lence, T. , Haussmann, I. U. , Jacob, D. , Kreim, N. , Carl, S. H. , … Roignant, J. Y. (2018). Zc3h13/Flacc is required for adenosine methylation by bridging the mRNA‐binding factor Rbm15/Spenito to the m(6)A machinery component Wtap/Fl(2)d. Genes & Development, 32(5–6), 415–429. 10.1101/gad.309146.117 29535189 PMC5900714

[wrna1489-bib-0058] Kohli, R. M. , & Zhang, Y. (2013). TET enzymes, TDG and the dynamics of DNA demethylation. Nature, 502(7472), 472–479. 10.1038/nature12750 24153300 PMC4046508

[wrna1489-bib-0059] Legrand, C. , Tuorto, F. , Hartmann, M. , Liebers, R. , Jacob, D. , Helm, M. , & Lyko, F. (2017). Statistically robust methylation calling for whole‐transcriptome bisulfite sequencing reveals distinct methylation patterns for mouse RNAs. Genome Research, 27(9), 1589–1596. 10.1101/gr.210666.116 28684555 PMC5580717

[wrna1489-bib-0060] Lenasi, T. , & Barboric, M. (2013). Mutual relationships between transcription and pre‐mRNA processing in the synthesis of mRNA. WIREs RNA, 4(2), 139–154. 10.1002/wrna.1148 23184646

[wrna1489-bib-0061] Lence, T. , Akhtar, J. , Bayer, M. , Schmid, K. , Spindler, L. , Ho, C. H. , … Roignant, J. Y. (2016). m6A modulates neuronal functions and sex determination in *Drosophila* . Nature, 540(7632), 242–247. 10.1038/nature20568 27919077

[wrna1489-bib-0062] Li, A. , Chen, Y. S. , Ping, X. L. , Yang, X. , Xiao, W. , Yang, Y. , … Yang, Y. G. (2017). Cytoplasmic m(6)A reader YTHDF3 promotes mRNA translation. Cell Research, 27(3), 444–447. 10.1038/cr.2017.10 28106076 PMC5339832

[wrna1489-bib-0063] Li, X. , Xiong, X. , Wang, K. , Wang, L. , Shu, X. , Ma, S. , & Yi, C. (2016). Transcriptome‐wide mapping reveals reversible and dynamic N(1)‐methyladenosine methylome. Nature Chemical Biology, 12(5), 311–316. 10.1038/nchembio.2040 26863410

[wrna1489-bib-0064] Li, X. , Xiong, X. , & Yi, C. (2016). Epitranscriptome sequencing technologies: Decoding RNA modifications. Nature Methods, 14(1), 23–31. 10.1038/nmeth.4110 28032622

[wrna1489-bib-0065] Li, X. , Xiong, X. , Zhang, M. , Wang, K. , Chen, Y. , Zhou, J. , … Yi, C. (2017). Base‐resolution mapping reveals distinct m(1)A methylome in nuclear‐ and mitochondrial‐encoded transcripts. Molecular Cell, 68(5), 993–1005 e1009. 10.1016/j.molcel.2017.10.019 29107537 PMC5722686

[wrna1489-bib-0066] Li, Z. , Weng, H. , Su, R. , Weng, X. , Zuo, Z. , Li, C. , … Chen, J. (2017). FTO plays an oncogenic role in acute myeloid leukemia as a N6‐methyladenosine RNA demethylase. Cancer Cell, 31(1), 127–141. 10.1016/j.ccell.2016.11.017 28017614 PMC5234852

[wrna1489-bib-0067] Lin, S. , Choe, J. , Du, P. , Triboulet, R. , & Gregory, R. I. (2016). The m(6)A methyltransferase METTL3 promotes translation in human cancer cells. Molecular Cell, 62(3), 335–345. 10.1016/j.molcel.2016.03.021 27117702 PMC4860043

[wrna1489-bib-0068] Linder, B. , Grozhik, A. V. , Olarerin‐George, A. O. , Meydan, C. , Mason, C. E. , & Jaffrey, S. R. (2015). Single‐nucleotide‐resolution mapping of m6A and m6Am throughout the transcriptome. Nature Methods, 12(8), 767–772. 10.1038/nmeth.3453 26121403 PMC4487409

[wrna1489-bib-0069] Liu, J. , Yue, Y. , Han, D. , Wang, X. , Fu, Y. , Zhang, L. , … He, C. (2014). A METTL3‐METTL14 complex mediates mammalian nuclear RNA N6‐adenosine methylation. Nature Chemical Biology, 10(2), 93–95. 10.1038/nchembio.1432 24316715 PMC3911877

[wrna1489-bib-0070] Liu, N. , Dai, Q. , Zheng, G. , He, C. , Parisien, M. , & Pan, T. (2015). N(6)‐methyladenosine‐dependent RNA structural switches regulate RNA‐protein interactions. Nature, 518(7540), 560–564. 10.1038/nature14234 25719671 PMC4355918

[wrna1489-bib-0071] Liu, N. , Parisien, M. , Dai, Q. , Zheng, G. , He, C. , & Pan, T. (2013). Probing N6‐methyladenosine RNA modification status at single nucleotide resolution in mRNA and long noncoding RNA. RNA, 19(12), 1848–1856. 10.1261/rna.041178.113 24141618 PMC3884656

[wrna1489-bib-0072] Liu, N. , Zhou, K. I. , Parisien, M. , Dai, Q. , Diatchenko, L. , & Pan, T. (2017). N6‐methyladenosine alters RNA structure to regulate binding of a low‐complexity protein. Nucleic Acids Research, 45(10), 6051–6063. 10.1093/nar/gkx141 28334903 PMC5449601

[wrna1489-bib-0073] Liu, S. R. , Hu, C. G. , & Zhang, J. Z. (2016). Regulatory effects of cotranscriptional RNA structure formation and transitions. WIREs RNA, 7(5), 562–574. 10.1002/wrna.1350 27028291

[wrna1489-bib-0074] Martinez‐Perez, M. , Aparicio, F. , Lopez‐Gresa, M. P. , Belles, J. M. , Sanchez‐Navarro, J. A. , & Pallas, V. (2017). Arabidopsis m6A demethylase activity modulates viral infection of a plant virus and the m6A abundance in its genomic RNAs. Proceedings of the National Academy of Sciences of the United States of America, 114(40), 10755–10760. 10.1073/pnas.1703139114 28923956 PMC5635872

[wrna1489-bib-0075] Mauer, J. , Luo, X. , Blanjoie, A. , Jiao, X. , Grozhik, A. V. , Patil, D. P. , … Jaffrey, S. R. (2017). Reversible methylation of m6Am in the 5′ cap controls mRNA stability. Nature, 541(7637), 371–375. 10.1038/nature21022 28002401 PMC5513158

[wrna1489-bib-0076] Merkestein, M. , Laber, S. , McMurray, F. , Andrew, D. , Sachse, G. , Sanderson, J. , … Cox, R. D. (2015). FTO influences adipogenesis by regulating mitotic clonal expansion. Nature Communications, 6, 6792 10.1038/ncomms7792 PMC4410642 25881961

[wrna1489-bib-0077] Meyer, K. D. , Patil, D. P. , Zhou, J. , Zinoviev, A. , Skabkin, M. A. , Elemento, O. , … Jaffrey, S. R. (2015). 5′ UTR m(6)A promotes cap‐independent translation. Cell, 163(4), 999–1010. 10.1016/j.cell.2015.10.012 26593424 PMC4695625

[wrna1489-bib-0078] Meyer, K. D. , Saletore, Y. , Zumbo, P. , Elemento, O. , Mason, C. E. , & Jaffrey, S. R. (2012). Comprehensive analysis of mRNA methylation reveals enrichment in 3′ UTRs and near stop codons. Cell, 149(7), 1635–1646. 10.1016/j.cell.2012.05.003 22608085 PMC3383396

[wrna1489-bib-0079] Molinie, B. , Wang, J. , Lim, K. S. , Hillebrand, R. , Lu, Z. X. , Van Wittenberghe, N. , … Giallourakis, C. C. (2016). m(6)A‐LAIC‐seq reveals the census and complexity of the m(6)A epitranscriptome. Nature Methods, 13(8), 692–698. 10.1038/nmeth.3898 27376769 PMC5704921

[wrna1489-bib-0080] Muthukrishnan, S. , Both, G. W. , Furuichi, Y. , & Shatkin, A. J. (1975). 5′‐Terminal 7‐methylguanosine in eukaryotic mRNA is required for translation. Nature, 255(5503), 33–37. 165427 10.1038/255033a0

[wrna1489-bib-0081] Nilsen, T. W. , & Graveley, B. R. (2010). Expansion of the eukaryotic proteome by alternative splicing. Nature, 463(7280), 457–463. 10.1038/nature08909 20110989 PMC3443858

[wrna1489-bib-0082] Ougland, R. , Zhang, C. M. , Liiv, A. , Johansen, R. F. , Seeberg, E. , Hou, Y. M. , … Falnes, P. O. (2004). AlkB restores the biological function of mRNA and tRNA inactivated by chemical methylation. Molecular Cell, 16(1), 107–116. 10.1016/j.molcel.2004.09.002 15469826

[wrna1489-bib-0083] Patil, D. P. , Chen, C. K. , Pickering, B. F. , Chow, A. , Jackson, C. , Guttman, M. , & Jaffrey, S. R. (2016). m(6)A RNA methylation promotes XIST‐mediated transcriptional repression. Nature, 537(7620), 369–373. 10.1038/nature19342 27602518 PMC5509218

[wrna1489-bib-0084] Pendleton, K. E. , Chen, B. , Liu, K. , Hunter, O. V. , Xie, Y. , Tu, B. P. , & Conrad, N. K. (2017). The U6 snRNA m6A methyltransferase METTL16 regulates SAM synthetase intron retention. Cell, 169(5), 824–835 e814. 10.1016/j.cell.2017.05.003 28525753 PMC5502809

[wrna1489-bib-0085] Perry, R. P. , & Kelley, D. E. (1974). Existence of methylated messenger‐RNA in mouse L cells. Cell, 1(1), 37–42. 10.1016/0092-8674(74)90153-6

[wrna1489-bib-0086] Ping, X. L. , Sun, B. F. , Wang, L. , Xiao, W. , Yang, X. , Wang, W. J. , … Yang, Y. G. (2014). Mammalian WTAP is a regulatory subunit of the RNA N6‐methyladenosine methyltransferase. Cell Research, 24(2), 177–189. 10.1038/cr.2014.3 24407421 PMC3915904

[wrna1489-bib-0087] Ramanathan, A. , Robb, G. B. , & Chan, S. H. (2016). mRNA capping: Biological functions and applications. Nucleic Acids Research, 44(16), 7511–7526. 10.1093/nar/gkw551 27317694 PMC5027499

[wrna1489-bib-0088] Roundtree, I. A. , Luo, G. Z. , Zhang, Z. , Wang, X. , Zhou, T. , Cui, Y. , … He, C. (2017). YTHDC1 mediates nuclear export of N6‐methyladenosine methylated mRNAs. eLife, 6 10.7554/eLife.31311 PMC5648532 28984244

[wrna1489-bib-0089] Ruzicka, K. , Zhang, M. , Campilho, A. , Bodi, Z. , Kashif, M. , Saleh, M. , … Fray, R. G. (2017). Identification of factors required for m6A mRNA methylation in Arabidopsis reveals a role for the conserved E3 ubiquitin ligase HAKAI. The New Phytologist, 215(1), 157–172. 10.1111/nph.14586 28503769 PMC5488176

[wrna1489-bib-0090] Safra, M. , Sas‐Chen, A. , Nir, R. , Winkler, R. , Nachshon, A. , Bar‐Yaacov, D. , … Schwartz, S. (2017). The m1A landscape on cytosolic and mitochondrial mRNA at single‐base resolution. Nature, 551(7679), 251–255. 10.1038/nature24456 29072297

[wrna1489-bib-0091] Salditt‐Georgieff, M. , Jelinek, W. , Darnell, J. E. , Furuichi, Y. , Morgan, M. , & Shatkin, A. (1976). Methyl labeling of HeLa cell hnRNA: A comparison with mRNA. Cell, 7(2), 227–237. 954080 10.1016/0092-8674(76)90022-2

[wrna1489-bib-0092] Saneyoshi, M. , Harada, F. , & Nishimura, S. (1969). Isolation and characterization of N6‐methyladenosine from *Escherichia coli* valine transfer RNA. Biochimica et Biophysica Acta, 190(2), 264–273. 4900574 10.1016/0005-2787(69)90078-1

[wrna1489-bib-0093] Schwartz, S. , Agarwala, S. D. , Mumbach, M. R. , Jovanovic, M. , Mertins, P. , Shishkin, A. , … Regev, A. (2013). High‐resolution mapping reveals a conserved, widespread, dynamic mRNA methylation program in yeast meiosis. Cell, 155(6), 1409–1421. 10.1016/j.cell.2013.10.047 24269006 PMC3956118

[wrna1489-bib-0094] Schwartz, S. , Mumbach, M. R. , Jovanovic, M. , Wang, T. , Maciag, K. , Bushkin, G. G. , … Regev, A. (2014). Perturbation of m6A writers reveals two distinct classes of mRNA methylation at internal and 5′ sites. Cell Reports, 8(1), 284–296. 10.1016/j.celrep.2014.05.048 24981863 PMC4142486

[wrna1489-bib-0095] Shimba, S. , Bokar, J. A. , Rottman, F. , & Reddy, R. (1995). Accurate and efficient N‐6‐adenosine methylation in spliceosomal U6 small nuclear RNA by HeLa cell extract in vitro. Nucleic Acids Research, 23(13), 2421–2426. 7630720 10.1093/nar/23.13.2421 PMC307046

[wrna1489-bib-0096] Sledz, P. , & Jinek, M. (2016). Structural insights into the molecular mechanism of the m(6)A writer complex. eLife, 5, e18434 10.7554/eLife.18434 27627798 PMC5023411

[wrna1489-bib-0097] Slobodin, B. , Han, R. , Calderone, V. , Vrielink, J. A. , Loayza‐Puch, F. , Elkon, R. , & Agami, R. (2017). Transcription impacts the efficiency of mRNA translation via co‐transcriptional N6‐adenosine methylation. Cell, 169(2), 326–337 e312. 10.1016/j.cell.2017.03.031 28388414 PMC5388891

[wrna1489-bib-0098] Spitale, R. C. , Flynn, R. A. , Zhang, Q. C. , Crisalli, P. , Lee, B. , Jung, J. W. , … Chang, H. Y. (2015). Structural imprints in vivo decode RNA regulatory mechanisms. Nature, 519(7544), 486–490. 10.1038/nature14263 25799993 PMC4376618

[wrna1489-bib-0099] Squires, J. E. , Patel, H. R. , Nousch, M. , Sibbritt, T. , Humphreys, D. T. , Parker, B. J. , … Preiss, T. (2012). Widespread occurrence of 5‐methylcytosine in human coding and non‐coding RNA. Nucleic Acids Research, 40(11), 5023–5033. 10.1093/nar/gks144 22344696 PMC3367185

[wrna1489-bib-0100] Stoltzfus, C. M. , & Dane, R. W. (1982). Accumulation of spliced avian retrovirus mRNA is inhibited in S‐adenosylmethionine‐depleted chicken embryo fibroblasts. Journal of Virology, 42(3), 918–931. 6285005 10.1128/jvi.42.3.918-931.1982 PMC256926

[wrna1489-bib-0101] Sundheim, O. , Vagbo, C. B. , Bjoras, M. , Sousa, M. M. , Talstad, V. , Aas, P. A. , … Slupphaug, G. (2006). Human ABH3 structure and key residues for oxidative demethylation to reverse DNA/RNA damage. EMBO Journal, 25(14), 3389–3397. 10.1038/sj.emboj.7601219 16858410 PMC1523172

[wrna1489-bib-0102] Tang, C. , Klukovich, R. , Peng, H. , Wang, Z. , Yu, T. , Zhang, Y. , … Yan, W. (2018). ALKBH5‐dependent m6A demethylation controls splicing and stability of long 3′‐UTR mRNAs in male germ cells. Proceedings of the National Academy of Sciences of the United States of America, 115(2), E325–E333. 10.1073/pnas.1717794115 29279410 PMC5777073

[wrna1489-bib-0103] Theler, D. , Dominguez, C. , Blatter, M. , Boudet, J. , & Allain, F. H. (2014). Solution structure of the YTH domain in complex with N6‐methyladenosine RNA: A reader of methylated RNA. Nucleic Acids Research, 42(22), 13911–13919. 10.1093/nar/gku1116 25389274 PMC4267619

[wrna1489-bib-0104] Tian, B. , Hu, J. , Zhang, H. , & Lutz, C. S. (2005). A large‐scale analysis of mRNA polyadenylation of human and mouse genes. Nucleic Acids Research, 33(1), 201–212. 10.1093/nar/gki158 15647503 PMC546146

[wrna1489-bib-0105] Tirumuru, N. , Zhao, B. S. , Lu, W. , Lu, Z. , He, C. , & Wu, L. (2016). N(6)‐methyladenosine of HIV‐1 RNA regulates viral infection and HIV‐1 Gag protein expression. Elife, 5 10.7554/eLife.15528 PMC4961459 27371828

[wrna1489-bib-0106] Topisirovic, I. , Svitkin, Y. V. , Sonenberg, N. , & Shatkin, A. J. (2011). Cap and cap‐binding proteins in the control of gene expression. WIREs RNA, 2(2), 277–298. 10.1002/wrna.52 21957010

[wrna1489-bib-0107] Tserovski, L. , Marchand, V. , Hauenschild, R. , Blanloeil‐Oillo, F. , Helm, M. , & Motorin, Y. (2016). High‐throughput sequencing for 1‐methyladenosine (m(1)A) mapping in RNA. Methods, 107, 110–121. 10.1016/j.ymeth.2016.02.012 26922842

[wrna1489-bib-0108] Wang, P. , Doxtader, K. A. , & Nam, Y. (2016). Structural basis for cooperative function of Mettl3 and Mettl14 methyltransferases. Molecular Cell, 63(2), 306–317. 10.1016/j.molcel.2016.05.041 27373337 PMC4958592

[wrna1489-bib-0109] Wang, X. , Feng, J. , Xue, Y. , Guan, Z. , Zhang, D. , Liu, Z. , … Yin, P. (2016). Structural basis of N(6)‐adenosine methylation by the METTL3–METTL14 complex. Nature, 534(7608), 575–578. 10.1038/nature18298 27281194

[wrna1489-bib-0110] Wang, X. , Lu, Z. , Gomez, A. , Hon, G. C. , Yue, Y. , Han, D. , … He, C. (2014). N6‐methyladenosine‐dependent regulation of messenger RNA stability. Nature, 505(7481), 117–120. 10.1038/nature12730 24284625 PMC3877715

[wrna1489-bib-0111] Wang, X. , Zhao, B. S. , Roundtree, I. A. , Lu, Z. , Han, D. , Ma, H. , … He, C. (2015). N(6)‐methyladenosine modulates messenger RNA translation efficiency. Cell, 161(6), 1388–1399. 10.1016/j.cell.2015.05.014 26046440 PMC4825696

[wrna1489-bib-0112] Wang, Y. , Li, Y. , Toth, J. I. , Petroski, M. D. , Zhang, Z. , & Zhao, J. C. (2014). N6‐methyladenosine modification destabilizes developmental regulators in embryonic stem cells. Nature Cell Biology, 16(2), 191–198. 10.1038/ncb2902 24394384 PMC4640932

[wrna1489-bib-0113] Warda, A. S. , Kretschmer, J. , Hackert, P. , Lenz, C. , Urlaub, H. , Hobartner, C. , … Bohnsack, M. T. (2017). Human METTL16 is a N6‐methyladenosine (m6A) methyltransferase that targets pre‐mRNAs and various non‐coding RNAs. EMBO Reports, 18, 2004–2014. https://doi.org/10.15252/embr.201744940 29051200 10.15252/embr.201744940 PMC5666602

[wrna1489-bib-0114] Wei, C. , Gershowitz, A. , & Moss, B. (1975a). N6, O2′‐dimethyladenosine a novel methylated ribonucleoside next to the 5′ terminal of animal cell and virus mRNAs. Nature, 257(5523), 251–253. 1161029 10.1038/257251a0

[wrna1489-bib-0115] Wei, C. M. , Gershowitz, A. , & Moss, B. (1975b). Methylated nucleotides block 5′ terminus of HeLa cell messenger RNA. Cell, 4(4), 379–386. 164293 10.1016/0092-8674(75)90158-0

[wrna1489-bib-0116] Wei, C. M. , Gershowitz, A. , & Moss, B. (1976). 5′‐Terminal and internal methylated nucleotide sequences in HeLa cell mRNA. Biochemistry, 15(2), 397–401. 174715 10.1021/bi00647a024

[wrna1489-bib-0117] Wei, C. M. , & Moss, B. (1975). Methylated nucleotides block 5′‐terminus of vaccinia virus messenger RNA. Proceedings of the National Academy of Sciences of the United States of America, 72(1), 318–322. 164018 10.1073/pnas.72.1.318 PMC432296

[wrna1489-bib-0118] Wei, C. M. , & Moss, B. (1977). Nucleotide sequences at the N6‐methyladenosine sites of HeLa cell messenger ribonucleic acid. Biochemistry, 16(8), 1672–1676. 856255 10.1021/bi00627a023

[wrna1489-bib-0119] Werner, M. , Purta, E. , Kaminska, K. H. , Cymerman, I. A. , Campbell, D. A. , Mittra, B. , … Bujnicki, J. M. (2011). 2′‐O‐ribose methylation of cap2 in human: Function and evolution in a horizontally mobile family. Nucleic Acids Research, 39(11), 4756–4768. 10.1093/nar/gkr038 21310715 PMC3113572

[wrna1489-bib-0120] Wilusz, J. E. , Freier, S. M. , & Spector, D. L. (2008). 3′ end processing of a long nuclear‐retained noncoding RNA yields a tRNA‐like cytoplasmic RNA. Cell, 135(5), 919–932. 10.1016/j.cell.2008.10.012 19041754 PMC2722846

[wrna1489-bib-0121] Wojtas, M. N. , Pandey, R. R. , Mendel, M. , Homolka, D. , Sachidanandam, R. , & Pillai, R. S. (2017). Regulation of m(6)A transcripts by the 3′→5′ RNA helicase YTHDC2 is essential for a successful meiotic program in the mammalian germline. Molecular Cell, 68(2), 374–387 e312. 10.1016/j.molcel.2017.09.021 29033321

[wrna1489-bib-0122] Xiang, Y. , Laurent, B. , Hsu, C. H. , Nachtergaele, S. , Lu, Z. , Sheng, W. , … Shi, Y. (2017). Corrigendum: RNA m(6)A methylation regulates the ultraviolet‐induced DNA damage response. Nature, 552(7685), 430 10.1038/nature24007 29186122

[wrna1489-bib-0123] Xiao, W. , Adhikari, S. , Dahal, U. , Chen, Y. S. , Hao, Y. J. , Sun, B. F. , … Yang, Y. G. (2016). Nuclear m(6)A reader YTHDC1 regulates mRNA splicing. Molecular Cell, 61(4), 507–519. 10.1016/j.molcel.2016.01.012 26876937

[wrna1489-bib-0124] Xu, L. , Liu, X. , Sheng, N. , Oo, K. S. , Liang, J. , Chionh, Y. H. , … Fu, X. Y. (2017). Three distinct 3‐methylcytidine (m(3)C) methyltransferases modify tRNA and mRNA in mice and humans. Journal of Biological Chemistry, 292(35), 14695–14703. 10.1074/jbc.M117.798298 28655767 PMC5582859

[wrna1489-bib-0125] Yang, X. , Yang, Y. , Sun, B. F. , Chen, Y. S. , Xu, J. W. , Lai, W. Y. , … Yang, Y. G. (2017). 5‐Methylcytosine promotes mRNA export—NSUN2 as the methyltransferase and ALYREF as an m5C reader. Cell Research, 27(5), 606–625. 10.1038/cr.2017.55 28418038 PMC5594206

[wrna1489-bib-0126] Yang, Y. , Fan, X. , Mao, M. , Song, X. , Wu, P. , Zhang, Y. , … Wang, Z. (2017). Extensive translation of circular RNAs driven by N6‐methyladenosine. Cell Research, 27(5), 626–641. 10.1038/cr.2017.31 28281539 PMC5520850

[wrna1489-bib-0127] Yue, Y. , Liu, J. , Cui, X. , Cao, J. , Luo, G. , Zhang, Z. , … Liu, J. (2018). VIRMA mediates preferential m(6)A mRNA methylation in 3′UTR and near stop codon and associates with alternative polyadenylation. Cell Discovery, 4, 10 10.1038/s41421-018-0019-0 29507755 PMC5826926

[wrna1489-bib-0128] Zhang, G. , Huang, H. , Liu, D. , Cheng, Y. , Liu, X. , Zhang, W. , … Chen, D. (2015). N6‐methyladenine DNA modification in *Drosophila* . Cell, 161(4), 893–906. 10.1016/j.cell.2015.04.018 25936838

[wrna1489-bib-0129] Zhang, S. , Zhao, B. S. , Zhou, A. , Lin, K. , Zheng, S. , Lu, Z. , … Huang, S. (2017). m6A demethylase ALKBH5 maintains tumorigenicity of glioblastoma stem‐like cells by sustaining FOXM1 expression and cell proliferation program. Cancer Cell, 31(4), 591–606 e596. 10.1016/j.ccell.2017.02.013 28344040 PMC5427719

[wrna1489-bib-0130] Zhao, B. S. , Wang, X. , Beadell, A. V. , Lu, Z. , Shi, H. , Kuuspalu, A. , … He, C. (2017). m6A‐dependent maternal mRNA clearance facilitates zebrafish maternal‐to‐zygotic transition. Nature, 542(7642), 475–478. 10.1038/nature21355 28192787 PMC5323276

[wrna1489-bib-0131] Zhao, X. , Yang, Y. , Sun, B. F. , Shi, Y. , Yang, X. , Xiao, W. , … Yang, Y. G. (2014). FTO‐dependent demethylation of N6‐methyladenosine regulates mRNA splicing and is required for adipogenesis. Cell Research, 24(12), 1403–1419. 10.1038/cr.2014.151 25412662 PMC4260349

[wrna1489-bib-0132] Zheng, G. , Dahl, J. A. , Niu, Y. , Fedorcsak, P. , Huang, C. M. , Li, C. J. , … He, C. (2013). ALKBH5 is a mammalian RNA demethylase that impacts RNA metabolism and mouse fertility. Molecular Cell, 49(1), 18–29. 10.1016/j.molcel.2012.10.015 23177736 PMC3646334

[wrna1489-bib-0133] Zhong, S. , Li, H. , Bodi, Z. , Button, J. , Vespa, L. , Herzog, M. , & Fray, R. G. (2008). MTA is an Arabidopsis messenger RNA adenosine methylase and interacts with a homolog of a sex‐specific splicing factor. Plant Cell, 20(5), 1278–1288. 10.1105/tpc.108.058883 18505803 PMC2438467

[wrna1489-bib-0134] Zhou, J. , Wan, J. , Gao, X. , Zhang, X. , Jaffrey, S. R. , & Qian, S. B. (2015). Dynamic m(6)A mRNA methylation directs translational control of heat shock response. Nature, 526(7574), 591–594. 10.1038/nature15377 26458103 PMC4851248

[wrna1489-bib-0135] Zhu, T. , Roundtree, I. A. , Wang, P. , Wang, X. , Wang, L. , Sun, C. , … Xu, Y. (2014). Crystal structure of the YTH domain of YTHDF2 reveals mechanism for recognition of N6‐methyladenosine. Cell Research, 24(12), 1493–1496. 10.1038/cr.2014.152 25412661 PMC4260350

[wrna1489-bib-0136] Zust, R. , Cervantes‐Barragan, L. , Habjan, M. , Maier, R. , Neuman, B. W. , Ziebuhr, J. , … Thiel, V. (2011). Ribose 2′‐O‐methylation provides a molecular signature for the distinction of self and non‐self mRNA dependent on the RNA sensor Mda5. Nature Immunology, 12(2), 137–143. 10.1038/ni.1979 21217758 PMC3182538

